# Supplementary material for: Fixed-dose ivermectin for Mass Drug Administration: Is it time to leave the dose pole behind? Insights from an Individual Participant Data Meta-Analysis
Source: PLoS Negl Trop Dis. 2025 Sep 15;19(9):e0013059. doi: 10.1371/journal.pntd.0013059 (PMC12449026; doi:10.1371/journal.pntd.0013059)
Supplement: S1 Table — (PDF) [file pntd.0013059.s001.pdf]

S1 Table: Summary of extracted data and sources for included studies

No.	Citation	Data Source	Access information	Data Extractor	Extracted Data	Date of Extraction
1	Demographic and Health Surveys (DHS) Program [1]	DHS Program	Registration for Dataset Access at: https://www.dhsprogram.com/data/dataset_admin/login_main.cfm Authorization confirmed by email to use the Survey Datasets of the requested countries.	AE	Age, Sex, Weight, Height and Study site of PSAC and WRA.	22/2/2024
2	Goss CW et al. (2019) [2]	Digital Commons@Becker data repository.	Data available for download and reuse at: https://digitalcommons.wustl.edu/	AE	Age, Sex, Weight, Height and Study site of SAC	14/8/24
3	Olola C et al (2020) [3]	Harvard Dataverse data repository	Available upon request from the corresponding author: Ph D. Christopher H O Olola (c_olola@yahoo.com)	AE	Age, Sex, Weight, Height and Study site of SAC	5/3/2024
4	Matamoros G et al. (2021) [4]	Dataset from study focused on soil-transmitted helminths (STH) interventions	Corresponding author: Gabriela Matamoros (gabriela.matamoros@unah.edu.hn) is author of the present study and provided dataset from previous study.	AE	Age, Sex, Weight, Height and Study site of SAC	25/8/2023
5	Krolewiecki A et al. (2025) [5]	Dataset from study focused on soil-transmitted helminths (STH) interventions	Corresponding author: Alejandro Krolewiecki (alekrol@mundosano.org) is author of the present study and provided dataset from previous study.	AE	Age, Sex, Weight, Height and Study site of SAC	22/6/2023
6	Echazú A et al. (2017)[6]	Dataset from study focused on soil-transmitted helminths (STH) interventions	Corresponding author: Adriana Echazú (adrianaechazu@hotmail.com) is author of the present study and provided dataset from previous study.	AE	Age, Sex, Weight, Height and Study site of PSAC and SAC	3/3/2023
7	Mwaiswelo RO et al. (2022) [7]	Infectious Diseases Data Observatory (IDDO)	Obtained under Data Use Agreement signed with Infectious Diseases Data Observatory (IDDO) (Agreement date: 21/3/2024). Access request at (www.iddo.org)	DB	Age, Sex, Weight, Height and Study site of PSAC and SAC	31/05/2024
8	COVID-19 data collected [8–12]	Infectious Diseases Data Observatory (IDDO)	Obtained under Data Use Agreement signed with Infectious Diseases Data Observatory (IDDO) (Agreement date: 21/3/2024). Access request at (www.iddo.org). COVID-19 data collected as part of the ALERRT consortium with the Clinical Characterization Protocol (part of ISARIC: https://isaric.org/research/covid-19-clinical-research-resources/clinical-characterisation-protocol-ccp/).	DB	Age, Sex, Weight, Height and Study site of PSAC and SAC	31/05/2024
9	Hamid et al. (2018) [13]	Infectious Diseases Data Observatory (IDDO)	Obtained under Data Use Agreement signed with Infectious Diseases Data Observatory (IDDO) (Agreement date: 21/3/2024). Access request at (www.iddo.org).	DB	Age, Sex, Weight, Height and Study site of PSAC and SAC	31/05/2024
10	Taylor WRJ et al. (2019) [14]	Infectious Diseases Data Observatory (IDDO)	Obtained under Data Use Agreement signed with Infectious Diseases Data Observatory (IDDO) (Agreement date: 21/3/2024). Access request at (www.iddo.org).	DB	Age, Sex, Weight, Height and Study site of PSAC and SAC	31/05/2024
11	Anvikar AR et al. (2012) [15]	Infectious Diseases Data Observatory (IDDO)	Obtained under Data Use Agreement signed with Infectious Diseases Data Observatory (IDDO) (Agreement date: 21/3/2024). Access request at (www.iddo.org).	DB	Age, Sex, Weight, Height and Study site of PSAC and SAC	31/05/2024
12	Silva M et al. (2019) [16]	Infectious Diseases Data Observatory (IDDO)	Obtained under Data Use Agreement signed with Infectious Diseases Data Observatory (IDDO) (Agreement date: 21/3/2024). Access request at (www.iddo.org).	DB	Age, Sex, Weight, Height and Study site of PSAC and SAC	31/05/2024
13	Carlsson AM (2011) [17]	Infectious Diseases Data Observatory (IDDO)	Obtained under Data Use Agreement signed with Infectious Diseases Data Observatory (IDDO) (Agreement date: 21/3/2024). Access request at (www.iddo.org).	DB	Age, Sex, Weight, Height and Study site of PSAC and SAC	31/05/2024

**14**	Mwaiswelo R (2019) [18]	Infectious Diseases Data Observatory (IDDO)	Obtained under Data Use Agreement signed with Infectious Diseases Data Observatory (IDDO) (Agreement date: 21/3/2024). Access request at (www.iddo.org).	DB	Age, Sex, Weight, Height and Study site of PSAC and SAC	31/05/2024
**15**	The Four Artemisinin-Based Combinations (4ABC) Study Group (2011)[19]	Infectious Diseases Data Observatory (IDDO)	Obtained under Data Use Agreement signed with Infectious Diseases Data Observatory (IDDO) (Agreement date: 21/3/2024). Access request at (www.iddo.org).	DB	Age, Sex, Weight, Height and Study site of PSAC and SAC	31/05/2024
**16**	Thriemer K et al. (2014) [20]	Infectious Diseases Data Observatory (IDDO)	Obtained under Data Use Agreement signed with Infectious Diseases Data Observatory (IDDO) (Agreement date: 21/3/2024). Access request at (www.iddo.org).	DB	Age, Sex, Weight, Height and Study site of PSAC and SAC	31/05/2024
**17**	Taylor WR et al. (2023) [21]	Infectious Diseases Data Observatory (IDDO)	Obtained under Data Use Agreement signed with Infectious Diseases Data Observatory (IDDO) (Agreement date: 21/3/2024). Access request at (www.iddo.org).	DB	Age, Sex, Weight, Height and Study site of PSAC and SAC	31/05/2024
**18**	Onyamboko MA et al. (2023) [22]	Infectious Diseases Data Observatory (IDDO)	Obtained under Data Use Agreement signed with Infectious Diseases Data Observatory (IDDO) (Agreement date: 21/3/2024). Access request at (www.iddo.org).	DB	Age, Sex, Weight, Height and Study site of PSAC and SAC	31/05/2024
**19**	Juma EA et al. (2008) [23]	Infectious Diseases Data Observatory (IDDO)	Obtained under Data Use Agreement signed with Infectious Diseases Data Observatory (IDDO) (Agreement date: 21/3/2024). Access request at (www.iddo.org).	DB	Age, Sex, Weight, Height and Study site of PSAC and SAC	31/05/2024
**20**	Abdallah TM et al. (2012) [24]	Infectious Diseases Data Observatory (IDDO)	Obtained under Data Use Agreement signed with Infectious Diseases Data Observatory (IDDO) (Agreement date: 21/3/2024). Access request at (www.iddo.org).	DB	Age, Sex, Weight, Height and Study site of PSAC and SAC	31/05/2024
**21**	Romani L et al. (2018) [25]	Infectious Diseases Data Observatory (IDDO)	Obtained under Data Use Agreement signed with Infectious Diseases Data Observatory (IDDO) (Agreement date: 21/3/2024). Access request at (www.iddo.org).	DB	Age, Sex, Weight, Height and Study site of PSAC and SAC	31/05/2024
**22**	Khalil EA et al. (2014) [26]	Infectious Diseases Data Observatory (IDDO)	Obtained under Data Use Agreement signed with Infectious Diseases Data Observatory (IDDO) (Agreement date: 21/3/2024). Access request at (www.iddo.org).	DB	Age, Sex, Weight, Height and Study site of PSAC and SAC	31/05/2024
**23**	Mueller Y et al. (2008) [27]	Infectious Diseases Data Observatory (IDDO)	Obtained under Data Use Agreement signed with Infectious Diseases Data Observatory (IDDO) (Agreement date: 21/3/2024). Access request at (www.iddo.org).	DB	Age, Sex, Weight, Height and Study site of PSAC and SAC	31/05/2024
**24**	Musa AM et al. (2010) [28]	Infectious Diseases Data Observatory (IDDO)	Obtained under Data Use Agreement signed with Infectious Diseases Data Observatory (IDDO) (Agreement date: 21/3/2024). Access request at (www.iddo.org).	DB	Age, Sex, Weight, Height and Study site of PSAC and SAC	31/05/2024
**25**	Cuypers B et al. (2018) [29]	Infectious Diseases Data Observatory (IDDO)	Obtained under Data Use Agreement signed with Infectious Diseases Data Observatory (IDDO) (Agreement date: 21/3/2024). Access request at (www.iddo.org).	DB	Age, Sex, Weight, Height and Study site of PSAC and SAC	31/05/2024
**26**	Rai K et al. (2017) [30]	Infectious Diseases Data Observatory (IDDO)	Obtained under Data Use Agreement signed with Infectious Diseases Data Observatory (IDDO) (Agreement date: 21/3/2024). Access request at (www.iddo.org).	DB	Age, Sex, Weight, Height and Study site of PSAC and SAC	31/05/2024
**27**	Imamura H et al. (2016) [31]	Infectious Diseases Data Observatory (IDDO)	Obtained under Data Use Agreement signed with Infectious Diseases Data Observatory (IDDO) (Agreement date: 21/3/2024). Access request at (www.iddo.org).	DB	Age, Sex, Weight, Height and Study site of PSAC and SAC	31/05/2024

**28**	Downing T et al. (2011) [32]	Infectious Diseases Data Observatory (IDDO)	Obtained under Data Use Agreement signed with Infectious Diseases Data Observatory (IDDO) (Agreement date: 21/3/2024). Access request at (www.iddo.org).	DB	Age, Sex, Weight, Height and Study site of PSAC and SAC	31/05/2024
**29**	Rijal S et al. (2010) [33]	Infectious Diseases Data Observatory (IDDO)	Obtained under Data Use Agreement signed with Infectious Diseases Data Observatory (IDDO) (Agreement date: 21/3/2024). Access request at (www.iddo.org).	DB	Age, Sex, Weight, Height and Study site of PSAC and SAC	31/05/2024
**30**	Rijal S et al. (2007) [34]	Infectious Diseases Data Observatory (IDDO)	Obtained under Data Use Agreement signed with Infectious Diseases Data Observatory (IDDO) (Agreement date: 21/3/2024). Access request at (www.iddo.org).	DB	Age, Sex, Weight, Height and Study site of PSAC and SAC	31/05/2024
**31**	Laurent T et al. (2006) [35]	Infectious Diseases Data Observatory (IDDO)	Obtained under Data Use Agreement signed with Infectious Diseases Data Observatory (IDDO) (Agreement date: 21/3/2024). Access request at (www.iddo.org).	DB	Age, Sex, Weight, Height and Study site of PSAC and SAC	31/05/2024
**32**	Yardley V (2005) [36]	Infectious Diseases Data Observatory (IDDO)	Obtained under Data Use Agreement signed with Infectious Diseases Data Observatory (IDDO) (Agreement date: 21/3/2024). Access request at (www.iddo.org).	DB	Age, Sex, Weight, Height and Study site of PSAC and SAC	31/05/2024
**33**	Sundar S et al. (2019) [37]	Infectious Diseases Data Observatory (IDDO)	Obtained under Data Use Agreement signed with Infectious Diseases Data Observatory (IDDO) (Agreement date: 21/3/2024). Access request at (www.iddo.org).	DB	Age, Sex, Weight, Height and Study site of PSAC and SAC	31/05/2024
**34**	Hailu A et al. (2010) [38]	Infectious Diseases Data Observatory (IDDO)	Obtained under Data Use Agreement signed with Infectious Diseases Data Observatory (IDDO) (Agreement date: 21/3/2024). Access request at (www.iddo.org).	DB	Age, Sex, Weight, Height and Study site of PSAC and SAC	31/05/2024
**35**	Bhattacharya SK et al. (2007) [39]	Infectious Diseases Data Observatory (IDDO)	Obtained under Data Use Agreement signed with Infectious Diseases Data Observatory (IDDO) (Agreement date: 21/3/2024). Access request at (www.iddo.org).	DB	Age, Sex, Weight, Height and Study site of PSAC and SAC	31/05/2024
**36**	Sundar S et al. (2010) [40]	Infectious Diseases Data Observatory (IDDO)	Obtained under Data Use Agreement signed with Infectious Diseases Data Observatory (IDDO) (Agreement date: 21/3/2024). Access request at (www.iddo.org).	DB	Age, Sex, Weight, Height and Study site of PSAC and SAC	31/05/2024
**37**	Pandey K et al. (2017) [41]	Infectious Diseases Data Observatory (IDDO)	Obtained under Data Use Agreement signed with Infectious Diseases Data Observatory (IDDO) (Agreement date: 21/3/2024). Access request at (www.iddo.org).	DB	Age, Sex, Weight, Height and Study site of PSAC and SAC	31/05/2024
**38**	Dysoley L et al. (2019) [42]	Infectious Diseases Data Observatory (IDDO)	Obtained under Data Use Agreement signed with Infectious Diseases Data Observatory (IDDO) (Agreement date: 21/3/2024). Access request at (www.iddo.org).	DB	Age, Sex, Weight, Height and Study site of PSAC and SAC	31/05/2024
**39**	Musa A et al. (2012) [43]	Infectious Diseases Data Observatory (IDDO)	Obtained under Data Use Agreement signed with Infectious Diseases Data Observatory (IDDO) (Agreement date: 21/3/2024). Access request at (www.iddo.org).	DB	Age, Sex, Weight, Height and Study site of PSAC and SAC	31/05/2024
**40**	Abreha T et al. (2018) [44]	Infectious Diseases Data Observatory (IDDO)	Obtained under Data Use Agreement signed with Infectious Diseases Data Observatory (IDDO) (Agreement date: 21/3/2024). Access request at (www.iddo.org).	DB	Age, Sex, Weight, Height and Study site of PSAC and SAC	31/05/2024
**41**	Sundar S et al. (2011) [45]	Infectious Diseases Data Observatory (IDDO)	Obtained under Data Use Agreement signed with Infectious Diseases Data Observatory (IDDO) (Agreement date: 21/3/2024). Access request at (www.iddo.org).	DB	Age, Sex, Weight, Height and Study site of PSAC and SAC	31/05/2024
**42**	Ley B et al. (2016) [46]	Infectious Diseases Data Observatory (IDDO)	Obtained under Data Use Agreement signed with Infectious Diseases Data Observatory (IDDO) (Agreement date: 21/3/2024). Access request at (www.iddo.org).	DB	Age, Sex, Weight, Height and Study site of PSAC and SAC	31/05/2024

**43**	Gonzalez-Ceron L et al. (2015) [47]	Infectious Diseases Data Observatory (IDDO)	Obtained under Data Use Agreement signed with Infectious Diseases Data Observatory (IDDO) (Agreement date: 21/3/2024). Access request at (www.iddo.org).	DB	Age, Sex, Weight, Height and Study site of PSAC46nd SAC	31/05/2024
**44**	Faucher JF et al. (2009) [48]	Infectious Diseases Data Observatory (IDDO)	Obtained under Data Use Agreement signed with Infectious Diseases Data Observatory (IDDO) (Agreement date: 21/3/2024). Access request at (www.iddo.org).	DB	Age, Weight, Height and Study site of PSAC and SAC	31/05/2024
**45**	Thuan PD et al. (2016) [49]	Infectious Diseases Data Observatory (IDDO)	Obtained under Data Use Agreement signed with Infectious Diseases Data Observatory (IDDO) (Agreement date: 21/3/2024). Access request at (www.iddo.org).	DB	Age, Sex, Weight, Height and Study site of SAC	31/05/2024
**46**	Adegbite BR et al. (2019) [50]	Infectious Diseases Data Observatory (IDDO)	Obtained under Data Use Agreement signed with Infectious Diseases Data Observatory (IDDO) (Agreement date: 21/3/2024). Access request at (www.iddo.org).	DB	Age, Sex, Weight, Height and Study site of SAC	31/05/2024
**47**	Pekyi D et al. (2016) [51]	Infectious Diseases Data Observatory (IDDO)	Obtained under Data Use Agreement signed with Infectious Diseases Data Observatory (IDDO) (Agreement date: 21/3/2024). Access request at (www.iddo.org).	DB	Age, Sex, Weight, Height and Study site of SAC	31/05/2024
**48**	Sundar S et al. (2008) [52]	Infectious Diseases Data Observatory (IDDO)	Obtained under Data Use Agreement signed with Infectious Diseases Data Observatory (IDDO) (Agreement date: 21/3/2024). Access request at (www.iddo.org).	DB	Age, Sex, Weight, Height and Study site of SAC	31/05/2024
**49**	Sundar S et al. (2009) [53]	Infectious Diseases Data Observatory (IDDO)	Obtained under Data Use Agreement signed with Infectious Diseases Data Observatory (IDDO) (Agreement date: 21/3/2024). Access request at (www.iddo.org).	DB	Age, Sex, Weight, Height and Study site of SAC	31/05/2024
**50**	Chakraborty D et al. (2008) [54]	Infectious Diseases Data Observatory (IDDO)	Obtained under Data Use Agreement signed with Infectious Diseases Data Observatory (IDDO) (Agreement date: 21/3/2024). Access request at (www.iddo.org).	DB	Age, Sex, Weight, Height and Study site of SAC	31/05/2024
**51**	Sundar S et al. (2014) [55]	Infectious Diseases Data Observatory (IDDO)	Obtained under Data Use Agreement signed with Infectious Diseases Data Observatory (IDDO) (Agreement date: 21/3/2024). Access request at (www.iddo.org).	DB	Age, Sex, Weight, Height and Study site of SAC	31/05/2024
**52**	Sundar S et al. (2015) [56]	Infectious Diseases Data Observatory (IDDO)	Obtained under Data Use Agreement signed with Infectious Diseases Data Observatory (IDDO) (Agreement date: 21/3/2024). Access request at (www.iddo.org).	DB	Age, Sex, Weight, Height and Study site of SAC	31/05/2024
**53**	Sundar S et al. (2011) [57]	Infectious Diseases Data Observatory (IDDO)	Obtained under Data Use Agreement signed with Infectious Diseases Data Observatory (IDDO) (Agreement date: 21/3/2024). Access request at (www.iddo.org).	DB	Age, Sex, Weight, Height and Study site of SAC	31/05/2024
**54**	Das VN et al. (2009) [58]	Infectious Diseases Data Observatory (IDDO)	Obtained under Data Use Agreement signed with Infectious Diseases Data Observatory (IDDO) (Agreement date: 21/3/2024). Access request at (www.iddo.org).	DB	Age, Sex, Weight, Height and Study site of SAC	31/05/2024
**55**	Pandey K et al. (2016) [59]	Infectious Diseases Data Observatory (IDDO)	Obtained under Data Use Agreement signed with Infectious Diseases Data Observatory (IDDO) (Agreement date: 21/3/2024). Access request at (www.iddo.org).	DB	Age, Sex, Weight, Height and Study site of SAC	31/05/2024
**56**	Sundar S et al. (2008) [60]	Infectious Diseases Data Observatory (IDDO)	Obtained under Data Use Agreement signed with Infectious Diseases Data Observatory (IDDO) (Agreement date: 21/3/2024). Access request at (www.iddo.org).	DB	Age, Sex, Weight, Height and Study site of SAC	31/05/2024
**57**	Sundar S et al. (2015) [61]	Infectious Diseases Data Observatory (IDDO)	Obtained under Data Use Agreement signed with Infectious Diseases Data Observatory (IDDO) (Agreement date: 21/3/2024). Access request at (www.iddo.org).	DB	Age, Sex, Weight, Height and Study site of SAC	31/05/2024

**58**	Sundar S et al. (2007) [62]	Infectious Diseases Data Observatory (IDDO)	Obtained under Data Use Agreement signed with Infectious Diseases Data Observatory (IDDO) (Agreement date: 21/3/2024). Access request at (www.iddo.org).	DB	Age, Sex, Weight, Height and Study site of SAC	31/05/2024
**59**	Sundar S et al. (2012) [63]	Infectious Diseases Data Observatory (IDDO)	Obtained under Data Use Agreement signed with Infectious Diseases Data Observatory (IDDO) (Agreement date: 21/3/2024). Access request at (www.iddo.org).	DB	Age, Sex, Weight, Height and Study site of SAC	31/05/2024
**60**	Sundar S et al. (2011) [64]	Infectious Diseases Data Observatory (IDDO)	Obtained under Data Use Agreement signed with Infectious Diseases Data Observatory (IDDO) (Agreement date: 21/3/2024). Access request at (www.iddo.org).	DB	Age, Sex, Weight, Height and Study site of SAC	31/05/2024

Note: Additionally, all individual datasets provided by IDDO will be nested under one main data package DOI, which will ensure that citations are properly linked and pulled through. Once the published article has a DOI or PubMed ID assigned the appropriate relationships will be established within the DOI metadata by the IDDO team.

References:

1. The DHS Program - Quality information to plan, monitor and improve population, health, and nutrition programs. [cited 10 Oct 2024]. Available: https://dhsprogram.com/

2. Goss CW, O'Brian K, Dubray C, Fischer PU, Hardy M, Jambulingam P, et al. Dosing pole recommendations for lymphatic filariasis elimination: A height-weight quantile regression modeling approach. PLoS Negl Trop Dis. 2019;13: e0007541. doi:10.1371/journal.pntd.0007541

3. Olola C, Agbenyega T, Kremsner PG, Newton CR, Bojang K, Taylor T. Data for: A multicenter, prospective observational study of intraleukocytic and intraerythrocytic pigment as prognostic features in African children with falciparum malaria. Harvard Dataverse; 2020. doi:10.7910/DVN/0CTWUJ

4. Matamoros G, Sánchez A, Gabrie JA, Juárez M, Ceballos L, Escalada A, et al. Efficacy and Safety of Albendazole and High-Dose Ivermectin Coadministration in School-Aged Children Infected With Trichuris trichiura in Honduras: A Randomized Controlled Trial. Clin Infect Dis Off Publ Infect Dis Soc Am. 2021;73: 1203–1210. doi:10.1093/cid/ciab365

5. Krolewiecki A, Kepha S, Fleitas PE, Lieshout L van, Gelaye W, Messa A, et al. Albendazole–ivermectin co-formulation for the treatment of Trichuris trichiura and other soil-transmitted helminths: a randomised phase 2/3 trial. Lancet Infect Dis. 2025;0. doi:10.1016/S1473-3099(24)00669-8

6. Echazú A, Juarez M, Vargas PA, Cajal SP, Cimino RO, Heredia V, et al. Albendazole and ivermectin for the control of soil-transmitted helminths in an area with high prevalence of Strongyloides stercoralis and hookworm in northwestern Argentina: A community-based pragmatic study. PLoS Negl Trop Dis. 2017;11: e0006003. doi:10.1371/journal.pntd.0006003

7. Mwaiswelo RO, Ngasala B, Msolo D, Kweka E, Mmbando BP, Mårtensson A. A single low dose of primaquine is safe and sufficient to reduce transmission of Plasmodium falciparum gametocytes regardless of cytochrome P450 2D6 enzyme activity in Bagamoyo district, Tanzania. Malar J. 2022;21: 84. doi:10.1186/s12936-022-04100-1

8. Centre d'Excellence Africain pour la Prévention, Gamal Abdel Nasser university of Conakry et le Contrôle des Maladies Transmissibles. COVID19 clinical data from Guinea. the Infectious Diseases Data Observatory; 2022. doi:10.48688/NUKD-1E1J

9. Institut Pasteur de Dakar. COVID19 clinical data from Senegal. the Infectious Diseases Data Observatory; 2020. doi:10.48688/FARH-S2PA

10. Lopez-Revilla JW. COVID 19 clinical data from the Instituto Nacional del Niño San Borja. the Infectious Diseases Data Observatory; 2021. doi:10.48688/YMRA-7280

11. Malawi-Liverpool-Wellcome Trust Clinical Research Programme. COVID 19 clinical data from the Malawi-Liverpool-Wellcome Trust Clinical Research Programme. the Infectious Diseases Data Observatory; 2020. doi:10.48688/ZD4R-YK37

12. National Institute for Communicable Diseases, South Africa. COVID 19 clinical data from the National Institute for Communicable Diseases, South Africa. the Infectious Diseases Data Observatory; 2020. doi:10.48688/6403-ZX05

13. Hamid MMA, Thriemer K, Elobied ME, Mahgoub NS, Boshara SA, Elsafi HMH, et al. Low risk of recurrence following artesunate-Sulphadoxine-pyrimethamine plus primaquine for uncomplicated Plasmodium falciparum and Plasmodium vivax infections in the Republic of the Sudan. Malar J. 2018;17: 117. doi:10.1186/s12936-018-2266-9

14. Taylor WRJ, Thriemer K, von Seidlein L, Yuentrakul P, Assawariyathipat T, Assefa A, et al. Short-course primaquine for the radical cure of Plasmodium vivax malaria: a multicentre, randomised, placebo-controlled non-inferiority trial. Lancet Lond Engl. 2019;394: 929–938. doi:10.1016/S0140-6736(19)31285-1

15. Anvikar AR, Sharma B, Shahi BH, Tyagi PK, Bose TK, Sharma SK, et al. Artesunate-amodiaquine fixed dose combination for the treatment of Plasmodium falciparum malaria in India. Malar J. 2012;11: 97. doi:10.1186/1475-2875-11-97

16. Silva M, Ferreira PE, Otienoburu SD, Calçada C, Ngasala B, Björkman A, et al. Plasmodium falciparum K13 expression associated with parasite clearance during artemisinin-based combination therapy. J Antimicrob Chemother. 2019;74: 1890–1893. doi:10.1093/jac/dkz098

17. Carlsson AM, Ngasala BE, Dahlström S, Membi C, Veiga IM, Rombo L, et al. Plasmodium falciparum population dynamics during the early phase of anti-malarial drug treatment in Tanzanian children with acute uncomplicated malaria. Malar J. 2011;10: 380. doi:10.1186/1475-2875-10-380

18. Mwaiswelo R, Ngasala B, Jovel I, Xu W, Larsson E, Malmberg M, et al. Prevalence of and Risk Factors Associated with Polymerase Chain Reaction-Determined Plasmodium falciparum Positivity on Day 3 after Initiation of Artemether-Lumefantrine Treatment for Uncomplicated Malaria in Bagamoyo District, Tanzania. Am J Trop Med Hyg. 2019;100: 1179–1186. doi:10.4269/ajtmh.18-0729

19. Four Artemisinin-Based Combinations (4ABC) Study Group. A head-to-head comparison of four artemisinin-based combinations for treating uncomplicated malaria in African children: a randomized trial. PLoS Med. 2011;8: e1001119. doi:10.1371/journal.pmed.1001119

20. Thriemer K, Hong NV, Rosanas-Urgell A, Phuc BQ, Ha DM, Pockele E, et al. Delayed parasite clearance after treatment with dihydroartemisinin-piperaquine in Plasmodium falciparum malaria patients in central Vietnam. Antimicrob Agents Chemother. 2014;58: 7049–7055. doi:10.1128/AAC.02746-14

21. Taylor WR, Olupot-Olupot P, Onyamboko MA, Peerawaranun P, Weere W, Namayanja C, et al. Safety of age-dosed, single low-dose primaquine in children with glucose-6-phosphate dehydrogenase deficiency who are infected with Plasmodium falciparum in Uganda and the Democratic Republic of the Congo: a randomised, double-blind, placebo-controlled, non-inferiority trial. Lancet Infect Dis. 2023;23: 471–483. doi:10.1016/S1473-3099(22)00658-2

22. Ma O, P O-O, W W, C N, P O, H T, et al. Factors affecting haemoglobin dynamics in African children with acute uncomplicated Plasmodium falciparum malaria treated with single low-dose primaquine or placebo. BMC Med. 2023;21. Available: https://www.ndm.ox.ac.uk/publications/1546798

23. Juma EA, Obonyo CO, Akhwale WS, Ogutu BR. A randomized, open-label, comparative efficacy trial of artemether-lumefantrine suspension versus artemether-lumefantrine tablets for treatment of uncomplicated Plasmodium falciparum malaria in children in western Kenya. Malar J. 2008;7: 262. doi:10.1186/1475-2875-7-262

24. Abdallah TM, Ali AAA, Bakri M, Gasim GI, Musa IR, Adam I. Efficacy of artemether-lumefantrine as a treatment for uncomplicated Plasmodium vivax malaria in eastern Sudan. Malar J. 2012;11: 404. doi:10.1186/1475-2875-11-404

25. Romani L, Marks M, Sokana O, Nasi T, Kamoriki B, Wand H, et al. Feasibility and safety of mass drug coadministration with azithromycin and ivermectin for the control of neglected tropical diseases: a single-arm intervention trial. Lancet Glob Health. 2018;6: e1132–e1138. doi:10.1016/S2214-109X(18)30397-8

26. Khalil EAG, Weldegebreal T, Younis BM, Omollo R, Musa AM, Hailu W, et al. Safety and efficacy of single dose versus multiple doses of AmBisome for treatment of visceral leishmaniasis in eastern Africa: a randomised trial. PLoS Negl Trop Dis. 2014;8: e2613. doi:10.1371/journal.pntd.0002613

27. Mueller Y, Nguimfack A, Cavailler P, Couffignal S, Rwakimari JB, Loutan L, et al. Safety and effectiveness of amphotericin B deoxycholate for the treatment of visceral leishmaniasis in Uganda. Ann Trop Med Parasitol. 2008;102: 11–19. doi:10.1179/136485908X252142

28. Musa AM, Younis B, Fadlalla A, Royce C, Balasegaram M, Wasunna M, et al. Paromomycin for the treatment of visceral leishmaniasis in Sudan: a randomized, open-label, dose-finding study. PLoS Negl Trop Dis. 2010;4: e855. doi:10.1371/journal.pntd.0000855

29. Cuypers B, Berg M, Imamura H, Dumetz F, De Muylder G, Domagalska MA, et al. Integrated genomic and metabolomic profiling of ISC1, an emerging Leishmania donovani population in the Indian subcontinent. Infect Genet Evol J Mol Epidemiol Evol Genet Infect Dis. 2018;62: 170–178. doi:10.1016/j.meegid.2018.04.021

30. Rai K, Bhattarai NR, Vanaerschot M, Imamura H, Gebru G, Khanal B, et al. Single locus genotyping to track Leishmania donovani in the Indian subcontinent: Application in Nepal. PLoS Negl Trop Dis. 2017;11: e0005420. doi:10.1371/journal.pntd.0005420

31. Imamura H, Downing T, Van den Broeck F, Sanders MJ, Rijal S, Sundar S, et al. Evolutionary genomics of epidemic visceral leishmaniasis in the Indian subcontinent. Soldati-Favre D, editor. eLife. 2016;5: e12613. doi:10.7554/eLife.12613

32. Downing T, Imamura H, Decuypere S, Clark T, Coombs G, Cotton J, et al. Whole genome sequencing of multiple Leishmania donovani clinical isolates provides insights into population structure and mechanisms of drug resistance. Genome Res. 2011;21: 2143–2156. doi:10.1101/gr.123430.111

33. Rijal S, Bhandari S, Koirala S, Singh R, Khanal B, Loutan L, et al. Clinical risk factors for therapeutic failure in kala-azar patients treated with pentavalent antimonials in Nepal. Trans R Soc Trop Med Hyg. 2010;104: 225–229. doi:10.1016/j.trstmh.2009.08.002

34. Rijal S, Yardley V, Chappuis F, Decuypere S, Khanal B, Singh R, et al. Antimonial treatment of visceral leishmaniasis: are current in vitro susceptibility assays adequate for prognosis of in vivo therapy outcome? Microbes Infect. 2007;9: 529–535. doi:10.1016/j.micinf.2007.01.009

35. Laurent T, Rijal S, Yardley V, Croft S, De Doncker S, Decuypere S, et al. Epidemiological dynamics of antimonial resistance in Leishmania donovani: genotyping reveals a polyclonal population structure among naturally-resistant clinical isolates from Nepal. Infect Genet Evol J Mol Epidemiol Evol Genet Infect Dis. 2007;7: 206–212. doi:10.1016/j.meegid.2006.08.005

36. Yardley V, Croft SL, De Doncker S, Dujardin J-C, Koirala S, Rijal S, et al. The sensitivity of clinical isolates of Leishmania from Peru and Nepal to miltefosine. Am J Trop Med Hyg. 2005;73: 272–275.

37. Sundar S, Singh A, Agrawal N, Chakravarty J. Effectiveness of Single-Dose Liposomal Amphotericin B in Visceral Leishmaniasis in Bihar. Am J Trop Med Hyg. 2019;101: 795–798. doi:10.4269/ajtmh.19-0179

38. Hailu A, Musa A, Wasunna M, Balasegaram M, Yifru S, Mengistu G, et al. Geographical Variation in the Response of Visceral Leishmaniasis to Paromomycin in East Africa: A Multicentre, Open-Label, Randomized Trial. PLoS Negl Trop Dis. 2010;4: e709. doi:10.1371/journal.pntd.0000709

39. Bhattacharya SK, Sinha PK, Sundar S, Thakur CP, Jha TK, Pandey K, et al. Phase 4 trial of miltefosine for the treatment of Indian visceral leishmaniasis. J Infect Dis. 2007;196: 591–598. doi:10.1086/519690

40. Sundar S, Chakravarty J, Agarwal D, Rai M, Murray HW. Single-dose liposomal amphotericin B for visceral leishmaniasis in India. N Engl J Med. 2010;362: 504–512. doi:10.1056/NEJMoa0903627

41. Pandey K, Pal B, Siddiqui NA, Rabi Das VN, Murti K, Lal CS, et al. Efficacy and Safety of Liposomal Amphotericin B for Visceral Leishmaniasis in Children and Adolescents at a Tertiary Care Center in Bihar, India. Am J Trop Med Hyg. 2017;97: 1498–1502. doi:10.4269/ajtmh.17-0094

42. Dysoley L, Kim S, Lopes S, Khim N, Bjorges S, Top S, et al. The tolerability of single low dose primaquine in glucose-6-phosphate deficient and normal falciparum-infected Cambodians. BMC Infect Dis. 2019;19: 250. doi:10.1186/s12879-019-3862-1

43. Musa A, Khalil E, Hailu A, Olobo J, Balasegaram M, Omollo R, et al. Sodium stibogluconate (SSG) & paromomycin combination compared to SSG for visceral leishmaniasis in East Africa: a randomised controlled trial. PLoS Negl Trop Dis. 2012;6: e1674. doi:10.1371/journal.pntd.0001674

44. Abreha T, Hwang J, Thriemer K, Tadesse Y, Girma S, Melaku Z, et al. Comparison of artemether-lumefantrine and chloroquine with and without primaquine for the treatment of Plasmodium vivax infection in Ethiopia: A randomized controlled trial. PLoS Med. 2017;14: e1002299. doi:10.1371/journal.pmed.1002299

45. Sundar S, Sinha PK, Verma DK, Kumar N, Alam S, Pandey K, et al. Ambisome plus miltefosine for Indian patients with kala-azar. Trans R Soc Trop Med Hyg. 2011;105: 115–117. doi:10.1016/j.trstmh.2010.10.008

46. Ley B, Alam MS, Thriemer K, Hossain MS, Kibria MG, Auburn S, et al. G6PD Deficiency and Antimalarial Efficacy for Uncomplicated Malaria in Bangladesh: A Prospective Observational Study. PLOS ONE. 2016;11: e0154015. doi:10.1371/journal.pone.0154015

47. Gonzalez-Ceron L, Rodriguez MH, Sandoval MA, Santillan F, Galindo-Virgen S, Betanzos AF, et al. Effectiveness of combined chloroquine and primaquine treatment in 14 days versus intermittent single dose regimen, in an open, non-randomized, clinical trial, to eliminate Plasmodium vivax in southern Mexico. Malar J. 2015;14: 426. doi:10.1186/s12936-015-0938-2

48. Faucher J-F, Aubouy A, Adeothy A, Cottrell G, Doritchamou J, Gourmel B, et al. Comparison of sulfadoxine-pyrimethamine, unsupervised artemether-lumefantrine, and unsupervised artesunate-amodiaquine fixed-dose formulation for uncomplicated plasmodium falciparum malaria in Benin: a randomized effectiveness noninferiority trial. J Infect Dis. 2009;200: 57–65. doi:10.1086/599378

49. Thuan, PD, Ca N, Van Toi, P, Nhien, NTT, Thanh, NV, Anh, ND, et al. A Randomized Comparison of Chloroquine Versus Dihydroartemisinin–Piperaquine for the Treatment of Plasmodium vivax Infection in Vietnam. Am J Trop Med Hyg. 2016;94: 879–885.

50. Adegbite BR, Edoa JR, Honkpehedji YJ, Zinsou FJ, Dejon-Agobe JC, Mbong-Ngwese M, et al. Monitoring of efficacy, tolerability and safety of artemether–lumefantrine and artesunate–amodiaquine for the treatment of uncomplicated Plasmodium falciparum malaria in Lambaréné, Gabon: an open-label clinical trial. Malar J. 2019;18: 424. doi:10.1186/s12936-019-3015-4

51. PREGACT Study Group, Pekyi D, Ampromfi AA, Tinto H, Traoré-Coulibaly M, Tahita MC, et al. Four Artemisinin-Based Treatments in African Pregnant Women with Malaria. N Engl J Med. 2016;374: 913–927. doi:10.1056/NEJMoa1508606

52. Sundar S, Rai M, Chakravarty J, Agarwal D, Agrawal N, Vaillant M, et al. New Treatment Approach in Indian Visceral Leishmaniasis: Single-Dose Liposomal Amphotericin B Followed by Short-Course Oral Miltefosine. Clin Infect Dis. 2008;47: 1000–1006. doi:10.1086/591972

53. Sundar S, Agrawal N, Arora R, Agarwal D, Rai M, Chakravarty J. Short-course paromomycin treatment of visceral leishmaniasis in India: 14-day vs 21-day treatment. Clin Infect Dis Off Publ Infect Dis Soc Am. 2009;49: 914–918. doi:10.1086/605438

54. Chakraborty D, Basu JM, Sen P, Sundar S, Roy S. Human placental extract offers protection against experimental visceral leishmaniasis: a pilot study for a phase-I clinical trial. Ann Trop Med Parasitol. 2008;102: 21–38. doi:10.1179/136485908X252133

55. Sundar S, Pandey K, Thakur CP, Jha TK, Das VNR, Verma N, et al. Efficacy and Safety of Amphotericin B Emulsion versus Liposomal Formulation in Indian Patients with Visceral Leishmaniasis: A Randomized, Open-Label Study. PLoS Negl Trop Dis. 2014;8: e3169. doi:10.1371/journal.pntd.0003169

56. Sundar S, Singh A, Chakravarty J, Rai M. Efficacy and safety of miltefosine in treatment of post-kala-azar dermal leishmaniasis. ScientificWorldJournal. 2015;2015: 414378. doi:10.1155/2015/414378

57. Sundar S, Sinha PK, Verma DK, Kumar N, Alam S, Pandey K, et al. Ambisome plus miltefosine for Indian patients with kala-azar. Trans R Soc Trop Med Hyg. 2011;105: 115–117. doi:10.1016/j.trstmh.2010.10.008

58. Das VNR, Siddiqui NA, Pandey K, Singh VP, Topno RK, Singh D, et al. A controlled, randomized nonblinded clinical trial to assess the efficacy of amphotericin B deoxycholate as compared to pentamidine for the treatment of antimony unresponsive visceral leishmaniasis cases in Bihar, India. Ther Clin Risk Manag. 2009;5: 117–124.

59. Pandey K, Ravidas V, Siddiqui NA, Sinha SK, Verma RB, Singh TP, et al. Pharmacovigilance of Miltefosine in Treatment of Visceral Leishmaniasis in Endemic Areas of Bihar, India. Am J Trop Med Hyg. 2016;95: 1100–1105. doi:10.4269/ajtmh.16-0242

60. S S, J C, D A, A S, N A, M R. Safety of a pre-formulated amphotericin B lipid emulsion for the treatment of Indian Kala-azar. Trop Med Int Health TM IH. 2008;13. doi:10.1111/j.1365-3156.2008.02128.x

61. Sundar S, Singh A, Rai M, Chakravarty J. Single-dose indigenous liposomal amphotericin B in the treatment of Indian visceral leishmaniasis: a phase 2 study. Am J Trop Med Hyg. 2015;92: 513–517. doi:10.4269/ajtmh.14-0259

62. Sundar S, Jha TK, Thakur CP, Sinha PK, Bhattacharya SK. Injectable paromomycin for Visceral leishmaniasis in India. N Engl J Med. 2007;356: 2571–2581. doi:10.1056/NEJMoa066536

63. Sundar S, Singh A, Rai M, Prajapati VK, Singh AK, Ostyn B, et al. Efficacy of miltefosine in the treatment of visceral leishmaniasis in India after a decade of use. Clin Infect Dis Off Publ Infect Dis Soc Am. 2012;55: 543–550. doi:10.1093/cid/cis474

64. Sundar S, Sinha PK, Rai M, Verma DK, Nawin K, Alam S, et al. Comparison of short-course multidrug treatment with standard therapy for visceral leishmaniasis in India: an open-label, non-inferiority, randomised controlled trial. The Lancet. 2011;377: 477–486. doi:10.1016/S0140-6736(10)62050-8
