# Supplementary material for: Fixed-dose ivermectin for Mass Drug Administration: Is it time to leave the dose pole behind? Insights from an Individual Participant Data Meta-Analysis
Source: PLoS Negl Trop Dis. 2025 Sep 15;19(9):e0013059. doi: 10.1371/journal.pntd.0013059 (PMC12449026; doi:10.1371/journal.pntd.0013059)
Supplement: S2 Table — (PDF) [file pntd.0013059.s002.pdf]

S2 Table: Nutritional assessment of children (2 to 15 years old) included in the study by region and by country. N= 343,324

Region/Country	Total number of children	Number of PSAC	Number of SAC	Stunted	Severity of malnutrition α
Sub Saharan Africa	99348 (28.9%)	87990 (88.6%)	11360 (11.4%)	29634 (32%)	High
Angola	3533	3533	0	1426 (40.4%)	Very high
Benin	119	110	9	ND	ND
Burkina Faso	514	429	85	174 (33.9%)	High
Cameroon	2539	2539	0	729 (28.7%)	Moderate
Chad	6114	6114	0	3192 (52.2%)	Very high
Congo	2477	2477	0	763 (30.8%)	High
Côte d'Ivoire	2995	2749	246	683 (24.8%)	Moderate
Democratic Republic of the Congo	5157	4795	362	2570 (49.8%)	Very high
Ethiopia	4616	3146	1470	1957 (45.9%)	Very high
Gabon	3829	3145	684	596 (18.7%)	Mild
Gambia	2905	2104	801	340 (16.2%)	Mild
Ghana	3498	2411	1087	445 (18.4%)	Mild
Guinea	2051	1990	61	613 (30%)	High
Kenya	12726	10101	2625	1770 (15.6%)	Mild
Liberia	1330	1330	0	463 (34.8%)	High
Madagascar	3292	3292	0	1333 (40.5%)	Very high
Malawi	4196	3189	1007	1112 (36.2%)	High
Mali	443	161	282	ND	ND
Mozambique	6697	5630	1067	2475 (37.1%)	High
Namibia	927	927	0	249 (26.9%)	Moderate
Nigeria	6985	6951	34	2673 (38.5%)	High
Rwanda	265	265	0	132 (49.8%)	Very high
Senegal	3146	3144	2	572 (18.2%)	Mild
Sierra Leone	2257	2257	0	748 (33.1%)	High
South Africa	1375	880	485	197 (19.6%)	Mild
Sudan	551	42	509	129 (24%)	Moderate
Tanzania	2941	2782	159	764 (28.1%)	Moderate
Uganda	3905	3539	366	1030 (26.4%)	Moderate
Zambia	5136	5127	9	1801 (35.1%)	High
Zimbabwe	2829	2829	0	696 (24.6%)	Moderate
West Asia	8066 (2.3%)	8066 (100%)	0	4401 (54.6%)	Very high
Yemen	8066	8066	0	4401 (54.6%)	Very high
South & Southeast Asia	193340 (56.3%)	185443 (95.9%)	7895 (4.1%)	76329 (39.8%)	High
Afganistan	159	21	138	0	Mild
Bangladesh	4562	4508	54	1416 (31.4%)	High
Cambodia	2247	2182	65	530 (23.6%)	Moderate
India	181932	176781	5151	73057 (40.4%)	Very high
Indonesia	1928	63	1865	670 (36.6%)	High

Laos People´s Democratic Republic	638	258	380	145 (23.2%)	Moderate
Nepal	1701	1615	86	492 (29.2%)	Moderate
Vietnam	160	15	145	15 (22.4%)	Moderate
Oceania	5779 (1.7%)	2798 (48.4%)	2981 (51.6%)	1424 (31.6%)	High
Fiji	1149	0	1149	9 (0.8%)	Mild
Papua New Guinea	3360	1980	1380	1415 (42.1%)	Very high
Solomon Islands	1270	818	452	ND	ND
Latin America & Caribbean	36791 (10.7%)	33104 (90%)	3687 (10%)	9007 (24.6%)	Moderate
Argentina	695	6	689	64 (10.4%)	Mild
Colombia	9696	9652	44	1431 (14.8%)	Mild
Dominican Republic	1868	1868	0	120 (6.4%)	Mild
Guatemala	7019	7019	0	3433 (48.9%)	Very high
Haiti	5765	3240	2525	933 (16.2%)	Mild
Honduras	5960	5656	304	1757 (29.6%)	Moderate
Mexico	55	9	46	10 (21.3%)	Moderate
Peru	5718	5654	64	11192 (21%)	Moderate
Other countries *	28	2	26	ND	
TOTAL	343324 (100%)	317401 (92.4%)	25923 (7.6%)	120718 (35.2%)	High

*: Bolivia, Brasil and Pakistan were grouped as other countries because too few participants from each country where available for inclusion. ND: no data available.
