# Supplementary material for: Fixed-dose ivermectin for Mass Drug Administration: Is it time to leave the dose pole behind? Insights from an Individual Participant Data Meta-Analysis
Source: PLoS Negl Trop Dis. 2025 Sep 15;19(9):e0013059. doi: 10.1371/journal.pntd.0013059 (PMC12449026; doi:10.1371/journal.pntd.0013059)
Supplement: S3 Table — Currently recommended wight-based and height-based and alternative age-based fixed-dose. (PDF) [file pntd.0013059.s003.pdf]

S3 Table: Ivermectin dosing regimens. Currently recommended wight-based and height-based and alternative age-based fixed-dose.

**IVM dosing regimen**	**Not currently recommended**	**3 mg**	**6 mg**	**9 mg**	**12 mg**	**15 mg**	**18 mg**
**Weight based**	**< 15 Kg**	**15 – 24 kg**	**25 – 35 kg**	**36 – 50 kg**	**51 – 65 kg**	**66 – 79 kg**	
**Height based**	**< 90 cm**	**90 – 119 cm**	**120 – 139 cm**	**140 – 159 cm**	**>159 cm**		
**Age-based fixed-dose**	**<2 years old ***	**PSAC**		**SAC**			**Adults**

*Note: No exclusion criteria was applied in this study. Participants included were older than 2 years old (24 months of age). Therefore, the results are not applicable for children younger than 2 years old.
