# Supplementary material for: Fixed-dose ivermectin for Mass Drug Administration: Is it time to leave the dose pole behind? Insights from an Individual Participant Data Meta-Analysis
Source: PLoS Negl Trop Dis. 2025 Sep 15;19(9):e0013059. doi: 10.1371/journal.pntd.0013059 (PMC12449026; doi:10.1371/journal.pntd.0013059)
Supplement: S4 Table — (PDF) [file pntd.0013059.s004.pdf]

S4 Table: List of contributors to the dataset requested to the IDDO Data Platform. All contributors provided consent for data sharing.

Institution Name	Contact Email
Oxford Clinical Research Unit - Vietnam	ekestelyn@oucru.org
Elizabeth Anyango Juma	jumaelizabeth@yahoo.com
Ric N Price	rprice@menzies.edu.au
Medicines for Malaria Venture (MMV)	borghinii@mmv.org
Glaucia Fernanda Cota	gcota@dndi.org
Lilia Gonzales-Ceron	lgonzal@insp.mx
Amelie Vantaux	amelie.vantaux@gmail.com
Catherine Falade	lillyfunke@yahoo.com
Joelle Brown	joelle.brown@ucsf.edu
Benedikt Ley	benedikt.ley@menzies.edu.au
Kamala Ley-Thriemer	kamala.ley-thriemer@menzies.edu.au
Harin Karunajeewa	Harin.Karunajeewa@wh.org.au
Charles King	Chk@case.edu
Stephanie Knopp	s.knopp@swisstph.ch
Jennifer Kaiser	Jennifer.keiser@swisstph.ch
Herve Menan	rvmenan@yahoo.fr
Maria Isabel Veiga	maria.isabel.veiga@gmail.com
Richard Mwaiselo	richiemwai@yahoo.com
Liwang Cui	liwangcui@usf.edu
Khadime Sylla	khadimesylla@yahoo.fr
Komal Raj Rijal	rijalkomal@gmail.com
Oumar Gaye	oumar.gaye@ucad.edu.sn
Chi Eziefula	c.eziefula@bsms.ac.uk
Umberto D'Alessandro	udalessandro@mrc.gm
Drug for Neglected Diseases Initiative	vgoyal@dndi.org
Anders Bjorkman	anders.bjorkman@ki.se
Issaka Sagara	isagara@icermali.org
Ayola Akim Adegnika	ayola-akim.adegnika@uni-tuebingen.de
Toure Andre Offianan	andre_offianan@yahoo.fr
Annette Erhart	aerhart@mrc.gm
Paritosh Malaviya	Paritosh.malaviya@yahoo.com
Simone S. Ladeia-Andrade	shawam@uol.com.br
Andre Siquiera	amsiqueira@gmail.com
Ishag Adam	ishagadam@hotmail.com
Lina Zuluaga-Iderraga	linazulu38@gmail.com
Teun Bousema	Teun.Bousema@radboudumc.nl
Fang Huang	ipdhuangfang@163.com
Kevin Kobylinski	kobylinskikevin@yahoo.com
Krishna Pandey	drkrishnapandey@yahoo.com
Kala-Azar Medical Research Centre	Paritosh_malaviya@yahoo.com
Jean Francois Faucher	jean-francois.faucher@unilim.fr
Gustavo Adolfo Sierra Romero	romgustavo@gmail.com
Harin Karunajeewa	Harin.Karunajeewa@wh.org.au
Jean-Claude Dujardin	jcdujardin@itg.be
Matthew Grigg	Matthew.grigg@menzies.edu.au
Niyamatali Siddiqui	niyamatalisiddiqui@yahoo.com
François Chappuis	francoischappuis@hcuge.ch
Abdoulaye A. Djimde	adjimde@icermali.org
José Luiz Fernandes Vieira	jvieira@ufpa.br
