# Supplementary figures and images for: Fixed-dose ivermectin for Mass Drug Administration: Is it time to leave the dose pole behind? Insights from an Individual Participant Data Meta-Analysis
Source: PLoS Negl Trop Dis. 2025 Sep 15;19(9):e0013059. doi: 10.1371/journal.pntd.0013059 (PMC12449026; doi:10.1371/journal.pntd.0013059)

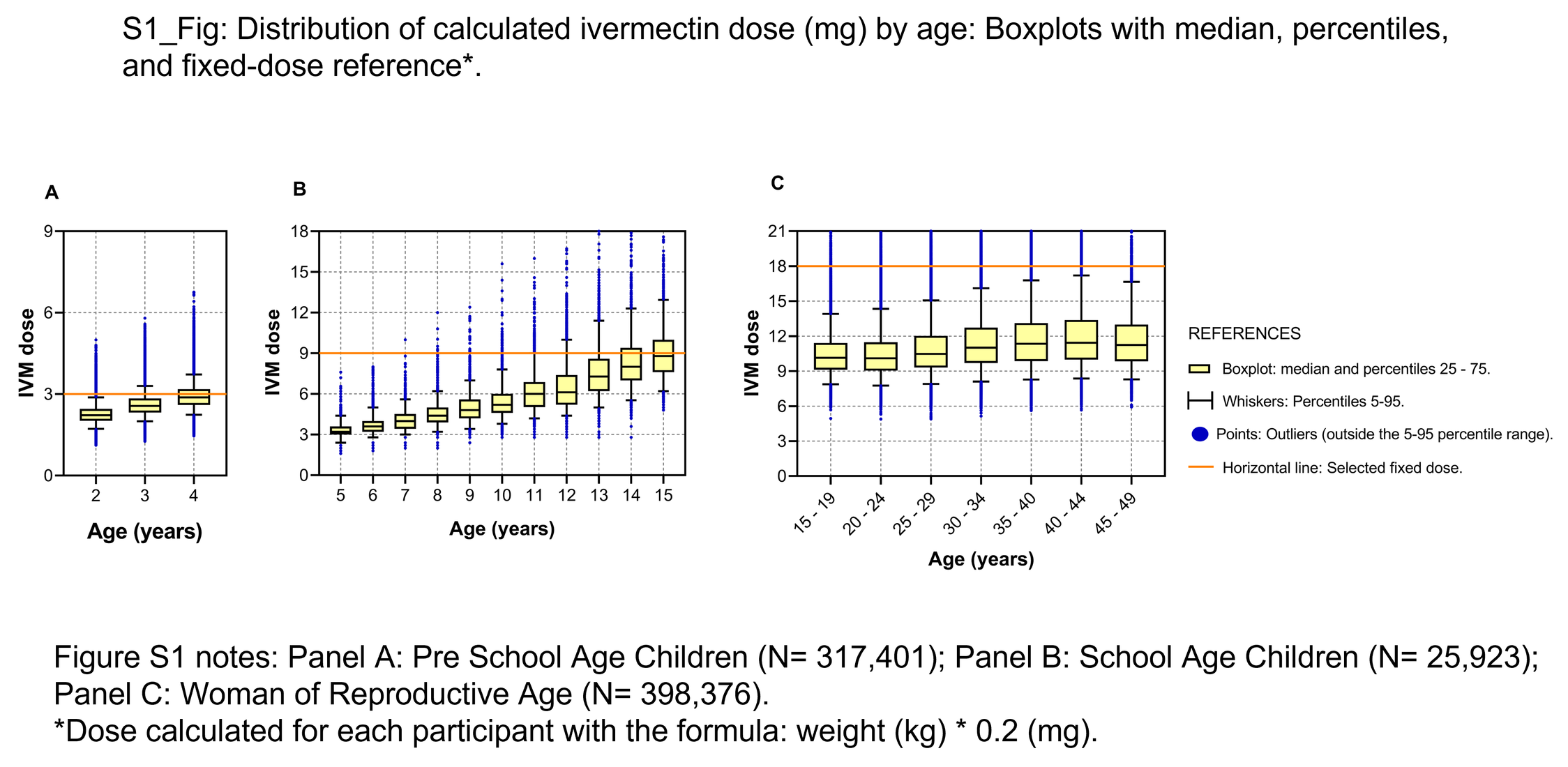

Supplement: S1 Fig — (TIF) [file pntd.0013059.s005.tif]

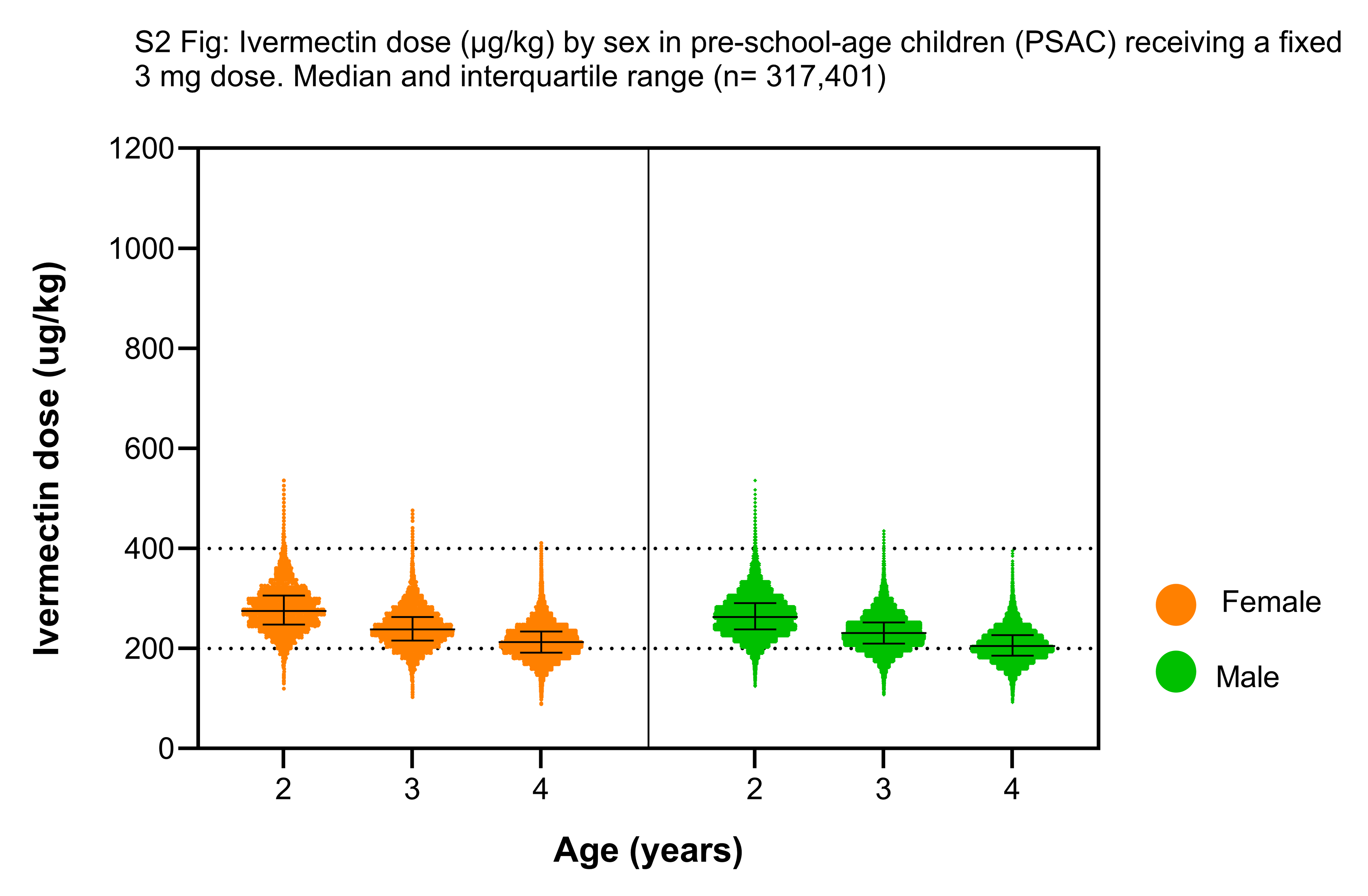

Supplement: S2 Fig — Median and interquartile range (n = 317,401). (TIF) [file pntd.0013059.s006.tif]

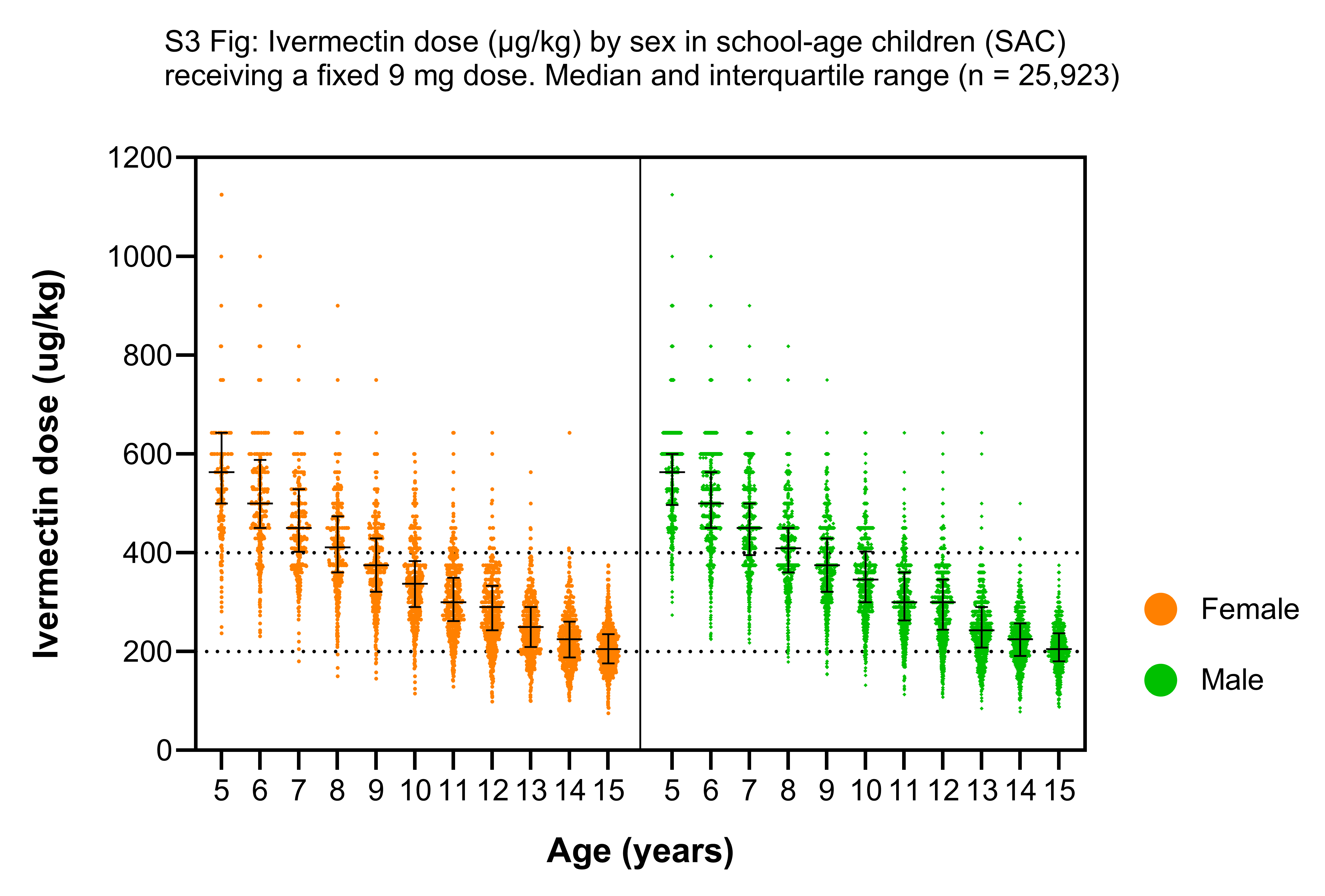

Supplement: S3 Fig — Median and interquartile range (n = 25,923). (TIF) [file pntd.0013059.s007.tif]
