# Supplementary material for: Fixed-dose ivermectin for Mass Drug Administration: Is it time to leave the dose pole behind? Insights from an Individual Participant Data Meta-Analysis
Source: PLoS Negl Trop Dis. 2025 Sep 15;19(9):e0013059. doi: 10.1371/journal.pntd.0013059 (PMC12449026; doi:10.1371/journal.pntd.0013059)
Supplement: S1 PRISMA Checklist — (PDF) [file pntd.0013059.s008.pdf]

PRISMA-IPD Checklist of items to include when reporting a systematic review and meta-analysis of individual participant data (IPD) [1]

PRISMA-IPD Section/topic	Item No	Checklist item	Reported on page
**Title**			
Title	1	Identify the report as a systematic review and meta-analysis of individual participant data.	1 (lines 1-2)
**Abstract**			
Structured summary	2	Provide a structured summary including as applicable:	Page 2
		**Background**: state research question and main objectives, with information on participants, interventions, comparators and outcomes.	Lines 22-26
		**Methods**: report eligibility criteria; data sources including dates of last bibliographic search or elicitation, noting that IPD were sought; methods of assessing risk of bias.	Lines 27-31
		**Results**: provide number and type of studies and participants identified and number (%) obtained; summary effect estimates for main outcomes (benefits and harms) with confidence intervals and measures of statistical heterogeneity. Describe the direction and size of summary effects in terms meaningful to those who would put findings into practice.	Lines 32-38
		**Discussion:** state main strengths and limitations of the evidence, general interpretation of the results and any important implications.	Lines 39-44
		**Other:** report primary funding source, registration number and registry name for the systematic review and IPD meta-analysis. PROSPERO registration	Lines 45-46
**Introduction**			
Rationale	3	Describe the rationale for the review in the context of what is already known.<br>**Ivermectin (IVM) is an essential medicine widely used in MDA activities**<br>**Current IVM dosing strategies pose operational challenges in large-scale MDA programs**<br>**height or weight-based dosing often leads to sub-therapeutic treatment**<br>**accumulating information on the safety of IVM at doses several times higher than currently recommended**	Lines 64-81
Objectives	4	Provide an explicit statement of the questions being addressed with reference, as applicable, to participants, interventions, comparisons, outcomes and study design (PICOS). Include any hypotheses that relate to particular types of participant-level subgroups.	Lines 83-85

		**This analysis aims to identify an alternative age-based dosing regimen using anthropometric data to evaluate fixed-dose regimens and compare drug exposure across current and exploratory strategies.**	
**Methods**			
Protocol and registration	5	Indicate if a protocol exists and where it can be accessed. If available, provide registration information including registration number and registry name. Provide publication details, if applicable.<br>**(PROSPERO 2024 CRD42024521610)**	Lines 90-92
Eligibility criteria	6	Specify inclusion and exclusion criteria including those relating to participants, interventions, comparisons, outcomes, study design and characteristics (e.g. years when conducted, required minimum follow-up). Note whether these were applied at the study or individual level i.e. whether eligible participants were included (and ineligible participants excluded) from a study that included a wider population than specified by the review inclusion criteria. The rationale for criteria should be stated.<br>**Studies were eligible if they included individual-level data on participants' anthropometric measurements and the location.**	Lines 100-101
Identifying studies - information sources	7	Describe all methods of identifying published and unpublished studies including, as applicable: which bibliographic databases were searched with dates of coverage; details of any hand searching including of conference proceedings; use of study registers and agency or company databases; contact with the original research team and experts in the field; open adverts and surveys. Give the date of last search or elicitation.<br>**Time frame: Studies conducted between January 1, 2010, and December 31, 2024.**<br>**IPD were obtained from two main sources: (1) studies on STH interventions conducted in endemic regions and (2) datasets from data repositories, accessed in compliance with their specific protocols.**	Line 106<br>Lines 121-123
Identifying studies - search	8	Present the full electronic search strategy for at least one database, including any limits used, such that it could be repeated.<br>**We requested data from IDDO with the following criteria: individual participant data from subjects older than 2 years, of any sex, with recorded weight, regardless of whether height was also recorded. The studies had to be conducted in countries endemic for soil-transmitted helminths (STH), and include outcomes related to malaria, STH and schistosomiasis, Chagas disease, or COVID-19. In response, IDDO identified 62 studies in their platforms that met these inclusion criteria and provided an anonymized, merged dataset containing the requested variables.**	

Study selection processes	9	State the process for determining which studies were eligible for inclusion.<br>**The datasets underwent a five-step process: selection, standardization, compilation, cleaning, and consistency assessment. First, only variables of interest were retained, excluding others from the original studies.**	Lines 124-128
Data collection processes	10	Describe how IPD were requested, collected and managed, including any processes for querying and confirming data with investigators. If IPD were not sought from any eligible study, the reason for this should be stated (for each such study).	Lines 121-123
		If applicable, describe how any studies for which IPD were not available were dealt with. This should include whether, how and what aggregate data were sought or extracted from study reports and publications (such as extracting data independently in duplicate) and any processes for obtaining and confirming these data with investigators.<br>**IPD were obtained from two main sources: (1) studies on STH interventions conducted in endemic regions and (2) datasets from data repositories, accessed in compliance with their specific protocols.**	
Data items	11	Describe how the information and variables to be collected were chosen. List and define all study level and participant level data that were sought, including baseline and follow-up information. If applicable, describe methods of standardising or translating variables within the IPD datasets to ensure common scales or measurements across studies.<br>**Study-Level Inclusion Criteria:**<br>**1. Study design: Observational studies, public health surveys, and clinical trials.**<br>**2. Geographic scope: Countries or sub-national districts where preventive chemotherapy for STH is recommended according to WHO guidelines [1,18].**<br>**3. Time frame: Studies conducted between January 1, 2010, and December 31, 2024.**<br>**4. Data availability: Studies including anthropometric data. Participants with missing or incomplete IPD for any mandatory variable (country, age, sex, and weight) were excluded. Individuals with missing height data were retained, as height was considered non-essential.**<br>**5. Timing of data collection: Only baseline data was included.**<br>**Study-Level Exclusion Criteria:**<br>**1. Studies providing only aggregated data.**<br>**2. Studies enrolling severely ill subjects, such as those focused on tuberculosis or severe malaria.**	Lines 102-119

		**Individual-Level Inclusion Criteria:**<br>**1. Age Groups: pre-school age children (PSAC): 2 to 4 years (24–59 months); school age children (SAC): 5 to 15 years; woman of reproductive age (WRA): 15 to 49 years.**<br>**2. Sex: Male and female (PSAC and SAC); only female adults (WRA)**<br><br>**Next, standardization ensured consistency across datasets: age was recorded in months (children) or years (adults), weight in kilograms (one decimal), and height in centimeters (one decimal). Each site was coded by country.**	Lines 126-128
IPD integrity	A1	Describe what aspects of IPD were subject to data checking (such as sequence generation, data consistency and completeness, baseline imbalance) and how this was done.<br>**Cleaning followed, removing subjects with missing data. Finally, a consistency assessment was conducted using WHO Anthro (version 3.2.2) and WHO Anthro Plus (version 1.0.4) to detect potential measurement errors**	Lines 131-134
Risk of bias assessment in individual studies.	12	Describe methods used to assess risk of bias in the individual studies and whether this was applied separately for each outcome. If applicable, describe how findings of IPD checking were used to inform the assessment. Report if and how risk of bias assessment was used in any data synthesis.<br>**Data completeness and consistency across studies were assessed, and participants with missing or inconsistent IPD were excluded according to pre-defined criteria applied systematically across all datasets.**<br><br>**To minimize measurement bias, data flagged as inconsistent by the software were excluded. However, this approach may introduce selection bias if exclusions were not proportionally distributed across sites or other characteristics.**	Lines 142-144<br><br>Lines 146-149
Specification of outcomes and effect measures	13	State all treatment comparisons of interests. State all outcomes addressed and define them in detail. State whether they were pre-specified for the review and, if applicable, whether they were primary/main or secondary/additional outcomes. Give the principal measures of effect (such as risk ratio, hazard ratio, difference in means) used for each outcome.<br>**The primary outcome of this study was: proportion of participants receiving target range dose of IVM (200 to 400 µg/kg) with a fixed-dose regimen based on age. A secondary outcome was to compare the performance of the fixed-dose**	Lines 198-204

		**regimen with height or weight-based dosing regimens. The measure of effect for this outcome were proportion and median doses differences between dosing regimens.**<br><br>**Prevalence of undernutrition in children globally and by country in the study population was another secondary outcome.**	
Synthesis methods	14	Describe the meta-analysis methods used to synthesise IPD. Specify any statistical methods and models used. Issues should include (but are not restricted to):<br><br>• Use of a one-stage or two-stage approach.<br>• How effect estimates were generated separately within each study and combined across studies (where applicable).<br>• Specification of one-stage models (where applicable) including how clustering of patients within studies was accounted for.<br>• Use of fixed or random effects models and any other model assumptions, such as proportional hazards.<br>• How (summary) survival curves were generated (where applicable).<br>• Methods for quantifying statistical heterogeneity (such as I^2 and τ^2).<br>• How studies providing IPD and not providing IPD were analysed together (where applicable).<br>• How missing data within the IPD were dealt with (where applicable).<br><br>**This study assumed population homogeneity, justifying a one-stage meta-analysis, and during the compilation step, IPD from different studies were merged into three datasets: PSAC, SAC, and WRA. Cleaning followed, removing subjects with missing data.**<br><br>**We applied a random effects model to address potential residual heterogeneity across the datasets included in the analysis and to examine the variation in effects across different subgroups. We assessed statistical heterogeneity using the I^2 statistic and τ^2, which allowed us to quantify the degree of variability.**	Lines 129-131<br><br>Lines 158-161
Exploration of variation in effects	A2	If applicable, describe any methods used to explore variation in effects by study or participant level characteristics (such as estimation of interactions between effect and covariates). State all participant-level characteristics that were analysed as potential effect modifiers, and whether these were pre-specified.	N/A
Risk of bias across studies	15	Specify any assessment of risk of bias relating to the accumulated body of evidence, including any pertaining to not obtaining IPD for particular studies, outcomes or other variables.	

		**The IPD compiled for this study were collected regardless of the design or outcomes of the original studies. Only baseline raw anthropometric IPD were included, rendering risks of bias related to outcome reporting or randomization processes irrelevant.**	Lines 140-142
Additional analyses	16	Describe methods of any additional analyses, including sensitivity analyses. State which of these were pre-specified.<br>**The proportion of participants in each dosing category (not currently recommended, undertreated, correctly treated, or above the target range) was calculated for each treatment regimen. To assess differences in proportions across regimens, pairwise comparisons with Chi-square test (α = 0.05) was performed. Additionally, median doses across the three regimens were compared using the Related Samples Friedman Test, stratified by group (PSAC, SAC, WRA). If significant differences were found, Wilcoxon signed-rank tests with Bonferroni correction were applied for post-hoc pairwise comparisons.**	Lines 190-196
**Results**			
Study selection and IPD obtained	17	Give numbers of studies screened, assessed for eligibility, and included in the systematic review with reasons for exclusions at each stage. Indicate the number of studies and participants for which IPD were sought and for which IPD were obtained. For those studies where IPD were not available, give the numbers of studies and participants for which aggregate data were available. Report reasons for non-availability of IPD. Include a flow diagram.<br>**Figure 1: Flow diagram of participants of the Individual participant data meta-analysis of IVM fixed-dose feasibility.**	Lines 231-232
Study characteristics	18	For each study, present information on key study and participant characteristics (such as description of interventions, numbers of participants, demographic data, unavailability of outcomes, funding source, and if applicable duration of follow-up). Provide (main) citations for each study. Where applicable, also report similar study characteristics for any studies not providing IPD.<br>**IPD were gathered from various original study datasets through the following sources: 1) Three studies focused on soil-transmitted helminths (STH) interventions conducted in Africa and Latin America [13,14,24]; 2) One study accessed via the Harvard Dataverse data repository [25]; 3) One dataset downloaded from the Digital Commons@Becker data repository [8]; 4) Datasets from 48 countries were requested to the Demographic and Health Surveys (DHS) Program [26]; and 5) Datasets from 62 studies were requested from the Infectious Diseases Data Observatory (IDDO)[27].**	Lines 216-222
IPD integrity	A3	Report any important issues identified in checking IPD or state that there were none.	Lines 222-225

		**During the data checking process, we identified some issues with the IPD: missing values for age or weight, inconsistent weight or height measurements, and formatting errors in age registration. These issues were addressed by excluding participants with any of these problems from the dataset.**	
Risk of bias within studies	19	Present data on risk of bias assessments. If applicable, describe whether data checking led to the up-weighting or down-weighting of these assessments. Consider how any potential bias impacts on the robustness of meta-analysis conclusions.<br>**Exclusions were performed randomly based on predefined criteria, and no significant differences were observed in the distribution of excluded participants across countries.**	Lines 225-227
Results of individual studies	20	For each comparison and for each main outcome (benefit or harm), for each individual study report the number of eligible participants for which data were obtained and show simple summary data for each intervention group (including, where applicable, the number of events), effect estimates and confidence intervals. These may be tabulated or included on a forest plot.<br>**N/A**	
Results of syntheses	21	Present summary effects for each meta-analysis undertaken, including confidence intervals and measures of statistical heterogeneity. State whether the analysis was pre-specified, and report the numbers of studies and participants and, where applicable, the number of events on which it is based.	Lines 239-242
		When exploring variation in effects due to patient or study characteristics, present summary interaction estimates for each characteristic examined, including confidence intervals and measures of statistical heterogeneity. State whether the analysis was pre-specified. State whether any interaction is consistent across trials.<br>**Heterogeneity analysis showed I^2 values of 17%, 18%, and 15% for PSAC, SAC, and WRA, respectively, indicating minimal heterogeneity. τ^2 values (0.005, 0.01, and 0.01) further confirmed low variability, suggesting that despite structural differences, heterogeneity across datasets was minimal.**	
		Provide a description of the direction and size of effect in terms meaningful to those who would put findings into practice.	
Risk of bias across studies	22	Present results of any assessment of risk of bias relating to the accumulated body of evidence, including any pertaining to the availability and representativeness of available studies, outcomes or other variables.	

Additional analyses	23	Give results of any additional analyses (e.g. sensitivity analyses). If applicable, this should also include any analyses that incorporate aggregate data for studies that do not have IPD. If applicable, summarise the main meta-analysis results following the inclusion or exclusion of studies for which IPD were not available.<br><br>**The distribution of weight by age was analyzed, revealing a homogeneous sample. Among PSAC, the mean weight was 12.93 (SD: 2.45) kg, with a median of 12.70 kg (IQR: 11.2–14.4). In SAC, the mean was 27.25 (SD: 11.08) kg, and the median 24.80 kg (IQR: 19–33). For WRA, the mean was 54.99 (SD: 11.96) kg, and the median 52.90 kg (IQR: 46.8–60.9).**<br><br>**The weight-based calculation identified a median IVM dose of 2.54 mg (IQR: 2.24–2.88) for PSAC; 4.96 mg (IQR: 3.8–6.6) for SAC; and 10.58 mg (IQR: 9.36–12.18) for WRA, required to achieve the dose of 200 µg/kg. Percentiles of the calculated IVM dose by year of age for children and by group for WRA are presented in Table 1 (also figure S2 in the Supporting information).**	Lines 248-255
**Discussion**			
Summary of evidence	24	Summarise the main findings, including the strength of evidence for each main outcome.<br><br>**This IPD meta-analysis, which included 741,700 participants from 53 NTD endemic countries, provides a comprehensive assessment of fixed-dose ivermectin regimens. By modelling drug exposure under different dosing strategies, our analysis demonstrates that an alternative age-based fixed-dose regimen achieves therapeutic dosing in a higher proportion of individuals compared to weight- and height-based regimens. Furthermore, we found that a simplified dosing approach reduces systematic underdosing without a great risk of exceeding established safety thresholds. This alternative approach, aligned with existing public health classification of at-risk groups (PSAC, SAC and WRA), could simplify drug administration logistics while contributing to dose optimization [1].**	Lines 337-347
Strengths and limitations	25	Discuss any important strengths and limitations of the evidence including the benefits of access to IPD and any limitations arising from IPD that were not available.<br><br>**A key strength of this study is the large number of participants, all from countries endemic for STH and other NTDs. The participants represent "real-world" recipients of MDA interventions, characterized by a high prevalence of malnutrition. As a result, evaluating fixed-dose IVM in this population enhances confidence in the low risk of excessive dosing. It is also relevant the homogeneity of the results across geographic regions.**<br><br>**This study has several limitations. SAC were underrepresented compared to PSAC and WRA, which may affect the generalizability of findings for this group. Despite SAC receiving the highest volume of anthelmintic drugs globally through school-based MDA programs targeting STH, anthropometric data remain scarce. Additionally, determining a**	Lines 367-387

		**fixed dose for SAC was challenging due to weight variation, as growth in this age range is steady. The selected dose prioritized minimizing underdosing.**<br><br>**The study relied on secondary data and the sources of IPD was diverse, which may have introduced inconsistencies or missing values, though heterogeneity analysis showed overall variability was low. Countries were assumed to be similar to be analyzed as a single population, but site-specific factors, such as India's unique nutritional and regulatory context, warrant further investigation of those sites, beyond the scope of this study.**<br><br>**Findings may also lack applicability to adult males, as no IPD were available for this group. Nonetheless, given that NTDs cause substantial morbidity in men and impact productivity in endemic regions, adult males would also likely benefit from IVM treatment [34,35]. The fixed dose for WRA was set at the upper weight range, ensuring adequate treatment and may also provide appropriate dosing to adult males.**	
Conclusions	26	Provide a general interpretation of the findings in the context of other evidence.<br><br>**In conclusion, the findings of this study provide robust evidence to inform policy discussions on IVM dosing, supporting the feasibility and benefits of transitioning from weight- and height-based IVM dosing to an age-based fixed-dose regimen. They offer critical insights into drug exposure among PSAC, a group currently excluded from MDA interventions. A fixed-dose strategy would reduce the substantial proportion of underdosed individuals without increasing the risk of toxicity [17].**	Lines 401-406
Implications	A4	Consider relevance to key groups (such as policy makers, service providers and service users). Consider implications for future research.<br><br>**Taken together, these findings present a compelling case as policy-makers at international, regional, and national levels consider updating treatment guidelines. The potential public health benefits -greater efficiency, broader coverage, and improved community engagement-underscore the importance of translating this evidence into action.**	Lines 407-410
**Funding**			
Funding	27	The study was conducted as part of a postdoctoral fellowship awarded to Adriana Echazu by Mundo Sano Foundation<br><br>**This research is part of the STOP2030 project, supported by the Global Health EDCTP3 Joint Undertaking and its members, and has received funding from the European Union's Horizon Europe research and innovation programme under grant agreement Nº 101103089. STOP2030 is also funded by the Federal Department of Economic Affairs, Education and Research (EAER) and the State Secretariat for Education, Research and Innovation (SERI) of the Swiss Confederation.**	

		**The funders had no role in study design, data collection/analysis, interpretation or the decision to submit the manuscript for publication.**	

A1 – A3 denote new items that are additional to standard PRISMA items. A4 has been created as a result of re-arranging content of the standard PRISMA statement to suit the way that systematic review IPD meta-analyses are reported.

[1] Page MJ, McKenzie JE, Bossuyt PM, Boutron I, Hoffmann TC, Mulrow CD, et al. The PRISMA 2020 statement: an updated guideline for reporting systematic reviews. BMJ 2021;372:n71. doi:10.1136/bmj.n71. Licensed under CC BY 4.0.

© Reproduced with permission of the PRISMA IPD Group, which encourages sharing and reuse for non-commercial purposes
