# Supplementary material for: Fixed-dose ivermectin for Mass Drug Administration: Is it time to leave the dose pole behind? Insights from an Individual Participant Data Meta-Analysis
Source: PLoS Negl Trop Dis. 2025 Sep 15;19(9):e0013059. doi: 10.1371/journal.pntd.0013059 (PMC12449026; doi:10.1371/journal.pntd.0013059)
Supplement: Attachment — Submitted filename: Response to reviewers.pdf [file pntd.0013059.s010.pdf]

Salta, Argentina, July 30, 2025

We thank the reviewers for their thoughtful comments, suggestions, and corrections, which have helped us improve the quality and clarity of our manuscript.

This document provides a point-by-point response to each of the reviewers' comments, presented in the order in which they appear in the review letters. Our responses follow each comment and are highlighted in blue for ease of reference. For comments that led to changes in the manuscript, we have indicated the corresponding line numbers where the revisions were made and included the modified text. Quoted text is highlighted in italics for easy reference.

Reviewers' Comments:

Reviewer #1: The Methods are well defined for a systematic review. It would be useful to clarify why men 15-49 were not included in the study, later in the Discussion it is explained that this data does not exist. But in the Methods line 119: "2. Sex: Male and female (PSAC and SAC); only female adults (WRA) [1]." it reads more like they were intentionally excluded from the review. Explaining earlier in the manuscript that this data does not exist would be useful for the reader.

Amended in line 123-124. *"Data for adult males were unavailable".* The exclusion of this group was primarily due to insufficient data, as our main source of individual participant data (DHS) does not collect height and weight information for adult men. Additionally, we focused on women of reproductive age (WRA) because they, along with preschool-aged children (PSAC) and school-aged children (SAC), are priority groups for mass drug administration (MDA) against soil-transmitted helminths (STH). Therefore, we aimed to highlight the performance of the proposed dosing regimen in this key target population, as is mentioned in line 377-378.

Line 181 should be updated as there appears to be Six groups of categorization?

Thank you for pointing this out. You are correct, there are six categories. The text has been updated accordingly.

-Are the objectives of the study clearly articulated with a clear testable hypothesis stated? YES

-Is the study design appropriate to address the stated objectives? YES

-Is the population clearly described and appropriate for the hypothesis being tested? See comment above

-Is the sample size sufficient to ensure adequate power to address the hypothesis being tested? YES

-Were correct statistical analysis used to support conclusions? YES

-Are there concerns about ethical or regulatory requirements being met? NO, this is clearly explained

Reviewer #2: The methods are appropriate to the objectives

Results

-Does the analysis presented match the analysis plan?

-Are the results clearly and completely presented?

-Are the figures (Tables, Images) of sufficient quality for clarity?

Reviewer #1:

-Does the analysis presented match the analysis plan? - YES

-Are the results clearly and completely presented? - YES

-Are the figures (Tables, Images) of sufficient quality for clarity? - Some improvements to the figures can be made, suggestions below.

Table 1 it looks as if the age 15 years is present both the SAC and WRA categories. Should WRA not start at 16 years? Especially since line 261 bases "median value for 15 yo for SAC" It would seem including this in both upper and lower bounds for two different categories causes a few more subjects being under- or over-dosed based?

The WRA category starts at 15 years of age in the WHO reference materials (https://www.who.int/data/gho/indicator-metadata-registry/imr-details/women-of-reproductive-age-(15-49-years)-population-(thousands)). We included 15-year-old participants in both the SAC and WRA groups because, for the SAC group, data were available for both sexes, whereas the WRA group included only females. Presenting this age in both categories allows readers to see that 15 years represents a threshold where both fixed doses of ivermectin (9 mg or 18 mg) are appropriate. This also illustrates that 15 years could be a practical cut-off age for transitioning between doses in the context of mass drug administration.

This was now better clarified within results section (lines 251-255):

"In accordance with WHO definitions, the WRA category begins at 15 years of age [30]. In this analysis, 15-year-old participants were included in both the SAC and WRA groups: in the SAC group to reflect data availability for both sexes, and in the WRA group, which included only females. Presenting this age in both categories highlights that 15 years could serve as a practical cut-off age for transitioning between fixed-dose regimens in MDA programs".

Figure 3, is it possible to label the panels? The Y-axis states "ugr/kg" and "microg/kgr". Neither of these are correct and should be "ug/kg" this is applied inconsistently throughout the manuscript, please update. Do not abbreviate axis labels. Can the figures be saved as vector based, they appear very pixelated even for the text.

Figures were provided in suboptimal quality in the initial submission to allow inclusion in a single PDF for easier review. All figures will be re-submitted as individual high-resolution vector files with the revised version. Y-axis label has been corrected according to your suggestion. The X-axis label remains abbreviated for clarity, but the abbreviation is now defined in the figure legend.

Figure 4, Y-axis needs to be clarified, Proportion of what? X-axis should be "Dosing Method" not "IVM dose regimen". Can the figures be saved as vector based, they appear very pixelated even for the text. Can the panels be labeled.

As per the author instructions of the journal, figure's number/title/caption must not be included within figure files. For this reason, the figure panels themselves are not labeled.

Figure 4 has been updated: both the Y-axis and X-axis have been re-labeled in accordance with your suggestions.

Figure 5, Panel A figure is really impactful and shows why the height and weight based dosing strategies are so misaligned, nice job! Is it possible to separate male and female subjects, perhaps in a separate figure? Panel B write out what 5, 50, 95 means. Panel C does not appear necessary to

extend to 800 ug/kg, but there is a tiny pixel floating perhaps where a 5 or 6 year old subject would be ??

Thank you for the kind feedback regarding Panel A.

For Panel B, a legend has been added to the figure, along with a footnote clarifying the meaning of the 5th, 50th, and 95th percentiles.

Regarding Panel C, thank you for pointing out the small floating point, this was indeed an error. In an earlier version of the figure, different colors were used to represent each year of age, and that point was part of the legend. While the legend was removed during revision, that element was mistakenly left behind. It does not correspond to an actual data point and has now been deleted.

A comment on differentiation between females and males is included in the next answer.

Can a similar breakout of age/dose by year be made for SAC?? Can the results for PSAC and SAC children be separated by sex? There are established differences in age/height/weight between boys and girls which could influence these dosing results. It would appear that your data set is large enough to assess this issue of sex differentiation.

We conducted an analysis to explore differences by sex; the findings are detailed in the paragraph below. Although some differences reached statistical significance, they do not appear to be clinically relevant, as there is no clear indication of a disproportionate risk of misdosing in one sex compared to the other with fixed-dose based on age. In fact, current ivermectin dosing strategies do not differentiate between boys and girls. The paragraph below was added to the results section to clarify this subject (lines 327-344)

"Sex differences

In the PSAC group, the median weight was 13 kg (IQR: 3.2 kg) for boys and 12.5 kg (IQR: 3.2 kg) for girls. A significant difference in weight distribution between sexes was observed, as confirmed by the Mann-Whitney U test ($p < 0.001$). In the SAC group, boys had a median weight of 24 kg (IQR: 13 kg), while girls had a median weight of 25 kg (IQR: 14 kg). A statistically significant difference was also identified in this group (Mann-Whitney U test, $p = 0.046$). In the PSAC group, 79.6% of boys and 83.8% of girls received the recommended dose of IVM with the fixed dose of 3 mg. Additionally, 0.2% of boys and 0.4% of girls received doses above the recommended range (200–400 µg/kg), with these differences being statistically significant (Fisher's exact test, $p < 0.001$). No child in the PSAC group, regardless of sex, received a dose above 600 µg/kg.

In the SAC group, 50.9% of boys and 51.5% of girls achieved the recommended dose using the fixed dose of 9 mg; this difference was not statistically significant (Fisher's exact test, $p = 0.154$). However, when analysing doses above the recommended (400 µg/kg), 42% of boys and 40% of girls exceeded this threshold with a statistically significant difference ($p = 0.039$). Within this group, only 0.5% of boys and 0.6% of girls received doses above 800 µg/kg, and this difference was not statistically significant ($p = 0.232$). Sex-based differences are presented in the supporting information (Figures S7 and S8)".

And this paragraph was added to the discussion section (lines 405-409):

"Regarding potential sex-based differences in dosing, our stratified analysis indicated that while some differences were statistically significant, they are unlikely to be clinically relevant. No clear pattern emerged suggesting a disproportionate risk of misdosing between males and females under age-based fixed-dose regimens. This finding aligns with current ivermectin dosing strategies, which do not differentiate between sexes".

Regarding figures I have added two new figures as supplementary material representing Ivermectin dose (ug/kg) by age with fixed-dose. Panel A: PSAC (3 mg); Panel B: SAC (9 mg). (S7 & S8)

Reviewer #2: There are some discrepancies with the PROSPERO registry. See below

Responded below.

Conclusions

Reviewer #1: -Are the conclusions supported by the data presented? YES

-Are the limitations of analysis clearly described? YES

-Do the authors discuss how these data can be helpful to advance our understanding of the topic under study? YES

-Is public health relevance addressed? YES

Citation #18 does not appear correct, #19 link is broken. Find a better citation to support the claim of 500 M people treated annually.

Citations are correct and links work properly to redirect to WHO web page to revise the number of doses of ivermectin distributed yearly for the control on onchorciasis and Lymphatic filariasis.

Reviewer #2: The conclusions are supported by data

Editorial and Data Presentation Modifications?

Use this section for editorial suggestions as well as relatively minor modifications of existing data that would enhance clarity. If the only modifications needed are minor and/or editorial, you may wish to recommend “Minor Revision” or “Accept”.

Reviewer #1: (No Response)

Reviewer #2: (No Response)

Summary and General Comments

Use this section to provide overall comments, discuss strengths/weaknesses of the study, novelty, significance, general execution and scholarship. You may also include additional comments for the author, including concerns about dual publication, research ethics, or publication ethics. If requesting major revision, please articulate the new experiments that are needed.

Reviewer #1: This is a very important manuscript that advances a critical concept to streamline ivermectin mass drug administration. The paper is clear and presents very useful comparison of weight-, height-, age- based fixed dosing.

Can the results for PSAC and SAC children be separated by sex? There are established differences in age/height/weight between boys and girls which could influence these dosing results. It would appear that your data set is large enough to assess this issue of sex differentiation.

We agree with this, please see above the details in the response to Reviewer#2. This analysis was performed and was now included as part of results section (lines 325-344) and discussion section (lines 405-409)

Reviewer #2: PNTD-D-25-00581

Fixed-Dose Ivermectin for Mass Drug Administration: Is it time to leave the dose pole behind? Insights from an Individual Participant Data Meta-Analysis. By Echazu et al.

This is a metanalysis conducted using individual patient data, evaluating the proportion of children in different age groups and women of reproductive age that receive the target dose of ivermectin based on anthropometric criteria. The authors propose fixed doses for the different target groups. A large dataset from countries endemic for STHs and filariae was used.

The manuscript is well written. The methods are appropriate for the objective and the conclusion is supported by the results.

Below I provide a few relevant comments

L75 “a clear upper safety limit for ivermectin in humans has not yet been formally established”

This is true but could be misleading to the non-expert reader. The studies of Kamgno some 20 years ago show good tolerability in adults but also a dose-dependent rate of AEs, particularly once the 800 µg/kg dose was exceeded. These AEs were not serious and indeed were mostly transient but could be the early signs of CNS toxicity. Buonfrate et al, saw a dose dependent AE rate when dosing either 600 or 1200 micrograms per kilo for five days. This is particularly worrisome for the neurologic category including Includes dizziness, headache, paraesthesia and somnolence.

Are these signs “safe”? that would be a good question for a regulatory agency, which should take into account the harm done by under-dosing in terms of target diseases and even potential resistance in the involved pathogens caused by sub-optimal systemic exposure. .

So, while the upper limit is not formally established, data seems to indicate the lower range is close to or at 800 µg/kg.

Thanks for these thoughts on the approaches for the understanding of the safety of ivermectin at high doses. Our statement that no safety limit has been established does not aim at leaving that upper limit open but rather as a call for attention that more data is needed. However, the data currently available, of which the safety analysis from Kamgno et al (TRSTMH, 2003) seems the richest, was done in participants with onchocerciasis and indeed identifies adverse events that were more related to the cycling and round of IVM doses than the dose itself; although, some adverse events were more frequent in the high-dose groups.

In order to prevent any misunderstanding, the paragraph was modified (and Kamgno´s reference added) and now states (lines 72-79):

“Early clinical trials evaluating the safety and efficacy of ivermectin for onchocerciasis demonstrated that single doses as high as 800 µg/kg were as safe as lower doses although some adverse events like edematous swelling and subjective ocular troubles were more frequent at doses of 800 µg/kg in subjects with onchocerciasis [9]. Notably, while these studies support the safety of higher doses, a clear upper safety limit for ivermectin in humans has not yet been formally established; therefore doses nearing 800 µg/kg should be approached with caution, especially when used for onchocerciasis”.

L197. Outcomes

There is a discrepancy with the PROSPERO registry which states: “Proportion of children receiving correct dose of ivermectin (200 to 600 µg/kg) with FDC compared with height or weight-based dosing”

The manuscript states 200-400

The PROSPERO registration was completed at an early stage of the study. Some adjustments were subsequently made as the study plan and preliminary results were presented at scientific meetings and discussed with experts. Initially, we intended to define the 'correct dose of ivermectin' as 200 to 600 µg/kg, based on evidence supporting the safe use of doses up to 600 µg/kg for certain indications, such as endectocidal for malaria control. However, we later chose to adopt a more conservative definition of the 'target dose range' as 200 to 400 µg/kg, aligning with formal WHO recommendations: 200 µg/kg for lymphatic filariasis and soil-transmitted helminths and 400 µg/kg for onchocerciasis.

L199. "A secondary outcome was to compare the performance of the fixed-dose regimen with height or weight-based dosing regimens. The measure of effect for this outcome were proportion and median doses differences between dosing regimens"

This is part of the primary in Prospero, not a secondary.

We aimed to shift the tone of the manuscript away from criticizing current dosing methods and instead focus on highlighting age-based dosing as a feasible alternative. For this reason, in the final version of the manuscript, the comparison with existing dosing approaches was moved to a secondary position.

What about the third outcome listed in PROSPERO? "Threshold age for contraindication of IVM".
What is the conclusion here?

That's a great question. According to our analysis, children aged 2 years and older—regardless of current contraindication criteria—could safely receive a 3 mg dose of ivermectin without exceeding the upper limit of 600 µg/kg. While we cannot definitively establish the minimum age for ivermectin administration, our findings suggest that existing contraindication criteria are inconsistent and not clearly based on the risk of overdosing. As a result, PSAC may be unnecessarily excluded from the potential benefits of ivermectin in controlling diseases that are common in this age group, such as soil-transmitted helminthiases and scabies.

Our conclusion is that our results of drug exposure by age correlate with the findings of the systematic review from Jittamala et al (cited in the Introduction) which identified safety profile in children weighing less than 15 kg comparable with children above that threshold weight.

We have incorporated this outcome into the methods section (lines 208-210) now states:

"Additional secondary outcomes were prevalence of undernutrition in children globally and by country in the study population and identification of a threshold age at which ivermectin administration may be contraindicated".

Within results the findings regarding this outcome where highlighted (lines 352-361):

"If a fixed 3 mg dose were administered to all PSAC without applying any anthropometric criteria for exclusion, the median dose delivered would be 236 µg/kg (IQR: 446 µg/kg). Under this approach, 81.1% (95% CI: 81.5–81.8) of children would receive a dose within the recommended range. Less than 0.3% (95% CI: 0.2–0.3) would exceed 400 µg/kg, and none would surpass 600 µg/kg".

"Regarding the threshold age for ivermectin administration, children typically reach 15 kg between 4 and 5 years of age (median weight: 14.4 kg at 4 years, 16 kg at 5 years). However, our findings suggest that children aged 2 years and older could receive a 3 mg dose without exceeding the 600 µg/kg upper limit, irrespective of current contraindication criteria".

In the discussion section this was also addressed as follows (lines 410-422):

"PSAC are systematically excluded from ivermectin treatment based on age, weight, or height criteria. However, these exclusion thresholds are inconsistently applied and not clearly aligned with the actual risk of overdosing. For example, age-based criteria often exclude children under five, even when their weight exceeds 15 kg [7]. Similarly, weight- and height-based regimens do not align with standard growth curves, resulting in poor concordance and the unnecessary exclusion of eligible children. This is especially concerning given the high burden of diseases like soil-transmitted helminthiases and scabies in PSAC, and their increased vulnerability to related morbidity. Our findings suggest that a fixed 3 mg dose would achieve adequate drug exposure in most PSAC, while addressing concerns about potential overdosing [13,37]. The observed drug exposure by age aligns with the systematic review by Jittamala et al., which found a comparable safety profile in children weighing below and above 15 kg [14]. Revisiting current exclusion criteria could prevent unnecessary barriers to treatment and support more equitable access of young children to ivermectin-based interventions".

L226 What do you mean by "Exclusions were performed randomly based on predefined criteria". Were data excluded at random?

Thank you for pointing this out. Te original sentence was unclear. Our intention was to clarify that the exclusions were based on predefined criteria, not random selection in the statistical sense. To ensure consistency and reliability, we applied predefined exclusion criteria (e.g., missing or implausible values for age, weight, or height) uniformly across the entire dataset. This process did not target or disproportionately affect any particular study or country. We have revised the paragraph in the manuscript to improve clarity on this point. (lines 229-237):

"During data checking, we identified issues in some individual records, such as missing values for age or weight, implausible height or weight measurements, or formatting errors in age registration. We addressed these problems by applying predefined exclusion criteria to remove affected participants from the dataset. These criteria were applied systematically across the full dataset and were not specific to any study, country, or subgroup. Importantly, the distribution of excluded participants was similar across countries, and no significant geographical selection bias was introduced. No entire study was excluded from the analysis; only individual participants were removed based on the criteria described.".

L279-281: "The proportion of participants classified as receiving doses above the recommended range was higher with the fixed-dose regimen (11.27%), a difference that was statistically significant compared to the other regimens"

These data (% receiving higher than recommended doses) should be presented

The proportion for the other dosing methods were presented in Table 2 (0% for weight-based and 0.003% for height-based). This information is now incorporated within the paragraph for clarity (lines 295-297):

"The proportion of participants classified as receiving doses above the recommended range was significantly higher with the fixed-dose regimen (11.27%) compared to the weight-based and height-based dosing methods, which had proportions of 0% and 0.003%, respectively".

L281. 0.02% of 500 million treated yearly is 100.000 exceeding 800 mcg/kg yearly. This should not be downplayed.

I can see your point, while this percentage may appear small, it represents a substantial number of individuals when scaled to the millions treated annually. We have rephrased the sentence to not to minimize this result. (lines 297-298):

"However, 0.02% exceeded the highest threshold (>800 µg/kg, level 3), with the maximum recorded dose reaching 1125 µg/kg".

Also a paragraph about safety concerns was added to the discussion section (lines 392-404), copied at the end of this letter.

L301. A version of figure 3 panel B but by category (PSAC; SAC and WRA) would be informative

We have created a new figure showing the fixed dose by age and by sex for children (S7 & S8), which we believe provides some of the details you requested as an alternative version of Figure 3, panel B.

L321-323 I don't quite see the "lack of concordance" as the pole limit of 90 cm effectively prevents approximately 95% of children under 15kg to receive treatment, hence fulfilling the current indication. It would be interesting however to quantify the proportion of children under 90cm who weight more than 15kg and are wrongly excluded from treatment using the pole.

A total of 1,271 (0.4%) children under 90 cm in height weighed more than 15 kg. The lack of concordance between the two methods arises because they exclude different children. Children with an average weight of 15 kg do not correspond to those with an average height of 90 cm; theoretically, 15 kg and 90 cm should align around the 50th percentile.

Discussion:

In general, is balanced and appropriate. It would be informative to include a paragraph on risk-management in those receiving higher doses.

Additional potentially important points:

What is the suggestion for children of uncertain age?

The authors report stunting rates exceeding 48% in some target countries. It would be valuable to mention/discuss the importance of protein binding and distribution of ivermectin in severely malnourished children. Is there any data regarding blood-brain barrier integrity and malnutrition?

Mentioning the case report from France of a child with a nonsensical mutation in the Pgp leading to an ICU admission could be useful as a reference to a rare event (PMID: 32813957)

Thank you for your suggestion. We have added an specific paragraph addressing safety concerns within discussion section (lines 392-404):

"While these findings support the feasibility of higher and age-based fixed-dose regimens, safety remains a key concern, particularly regarding the potential risks associated with exceeding recommended dose thresholds. Although ivermectin does not normally cross the blood–brain barrier, there is a theoretical risk of increased permeability in individuals with severe malnutrition, potentially elevating the risk of central nervous system toxicity. Despite the safety profile of ivermectin is well established, with millions of doses administered worldwide, serious neurological events have been reported, mostly in individuals with high-intensity Loa loa infections or during treatment for onchocerciasis [34]. Additionally, rare genetic mutations affecting drug transport mechanisms can predispose individuals to severe adverse effects and could become more relevant in large-scale MDA campaigns [35]. These considerations highlight the importance of including robust pharmacovigilance systems (both active and passive) as an essential component of any intervention using fixed-dose strategies based on age, despite the logistical and financial challenges they may entail".

Data accessibility:

I could not access the data at DOI: 10.6084/m9.figshare.28734971.

The DOI provided by figshare seems to be a common one for the site. If you direct to the link provided (the one copied above) and write Echazu in the Browse section you will find the project file and the datasets and corresponding manuals within.

Otherwise you can use this longer link that goes directly to the file: https://figshare.com/articles/dataset/_b_Fixed-Dose_Ivermectin_for_Mass_Drug_Administration_Is_it_time_to_leave_the_dose_pole_behind_Insights_from_an_Individual_Participant_Data_Meta-Analysis_b_/28734971

PLOS authors have the option to publish the peer review history of their article (what does this mean?). If published, this will include your full peer review and any attached files.

If you choose "no", your identity will remain anonymous but your review may still be made public.

Do you want your identity to be public for this peer review? For information about this choice, including consent withdrawal, please see our Privacy Policy.

Reviewer #1: No

Reviewer #2: No
